# Beyond the Known and Established Neurodegenerative Effects: Roles of APOE Across a Wide Spectrum of Pathophysiological Condition

**DOI:** 10.1002/mco2.70789

**Published:** 2026-06-04

**Authors:** Miriam Frosina, Samantha Maurotti, Alberto Castagna, Tiziana Montalcini, Arturo Pujia, Luca Tirinato

**Affiliations:** ^1^ Department of Medical and Surgical Sciences Magna Græcia University Catanzaro Italy; ^2^ Department of Clinical and Experimental Medicine Magna Græcia University Catanzaro Italy

**Keywords:** apolipoprotein E, bone mineral density, cardiovascular system, hepatic system, muscle mass, obesity

## Abstract

Apolipoprotein E (ApoE) is classically recognized for its role in lipid trafficking and the coordination of lipoprotein metabolism, yet its influence extends well beyond these pathways. While the contribution of ApoE isoforms to neurodegenerative disorders, most notably Alzheimer's disease, has been described in considerable detail, their impact on peripheral physiology is far less clearly defined. Evidence accumulated over the past decade suggests that variation in ApoE may shape traits such as adiposity, fat and lean mass distribution, bone density, muscle function, and cardiovascular risk, although the findings are often inconsistent across studies and populations. This review brings together current knowledge on how ApoE interfaces with several key biological processes, including inflammatory signaling, glucose and insulin responses, mitochondrial and redox homeostasis, senescence, and regulated cell death. These pathways lie at the core of many chronic disorders, yet their links to ApoE genotype remain insufficiently defined. Moreover, translation of these findings, including the use of ApoE genotyping for risk stratification, therapeutic choices, and personalized prevention is also discussed. By reframing ApoE as a systemic regulator rather than a brain‐restricted factor, this review offers a cohesive roadmap for interdisciplinary research and improved clinical interpretability of ApoE‐associated risk.

## Graphical abstract

APOE is widely recognized for its role in neurodegeneration and cardiovascular disease, but its different genotypes also influence body composition, affecting obesity, bone density, and muscle mass, through their central role in lipid transport and metabolism.

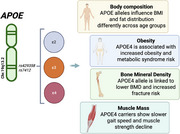

## 1 Introduction

Apolipoprotein E (ApoE) is a secreted, ∼34 kDa amphipathic glycoprotein that associates with plasma and central nervous system (CNS) lipoproteins to regulate lipid redistribution and receptor‐mediated lipoprotein clearance. It entered the scientific panorama in the early 1970s, when Shore described it as a component of very low‐density lipoproteins (VLDLs) [1]. At that time, it was referred to simply as an “arginine‐rich apoprotein,” but a few years later, Utermann proposed the name that is still in use today, recognizing it as a distinct member of the apolipoprotein family [2]. Subsequent milestone discoveries established common APOE isoforms (E2, E3, E4) and their biochemical basis, classically attributed to substitutions at residues 112 and 158, with major implications for lipoprotein handling and disease susceptibility. Since that initial characterization, interest in ApoE has grown steadily, largely because this small amphipathic protein appears in almost every vertebrate species examined, from mammals to fish, suggesting strong evolutionary pressure to maintain its structure and function [3]. In humans, ApoE comprises two interacting domains: an N‐terminal (NT) four‐helix bundle that contains the receptor‐binding region (supporting interactions with members of the LDL receptor family) and a C‐terminal (CT) lipid‐binding domain that mediates lipoprotein association and self‐assembly [4]. This structure underpins its main physiological task: moving cholesterol and phospholipids between tissues.

What once seemed a relatively straightforward lipid‐transport protein is now recognized as a far more versatile player. As research expanded, it became clear that ApoE plays a role in processes that at first glance appear to be unrelated to lipid biology. These range from immune signaling and inflammatory responses to mitochondrial regulation, glucose handling, and longevity pathways. This broad involvement across systems has raised the possibility that several common disorders, previously thought to be distinct, may share mechanistic roots that converge on ApoE activity or isoform status.

The ε4 allele of APOE is best known for its strong association with Alzheimer's disease (AD). This discovery fueled decades of work on the brain‐related roles of ApoE and firmly established it as a major determinant of neurodegenerative risk. Cardiovascular studies soon followed, revealing equally important effects on atherosclerosis and lipoprotein metabolism. By contrast, much less effort has been devoted to understanding how ApoE influences the development of other highly prevalent conditions such as obesity, the distribution of fat and lean mass, osteoporosis, or sarcopenia, even though the underlying pathways involve metabolic and inflammatory circuits in which ApoE is already known to participate.

Given the emerging view of ApoE as a systemic regulator, revisiting this protein from a broader biological perspective is timely. ApoE interacts with insulin and glucose‐regulated pathways, modulates oxidative stress responses, influences mitochondrial quality control, and participates in tissue repair and immune regulation. These functions operate across multiple organs, meaning that ApoE biology is likely to be relevant to a much wider spectrum of diseases than traditionally appreciated. Recent advances in genomics, lipidomics, single‐cell analyses, and targeted therapeutics have placed ApoE at the center of several emerging research fields, with clear implications for personalized and preventive medicine.

For these reasons, in this review, we examine ApoE from structure to function and consider how isoform‐specific biology may influence chronic metabolic and musculoskeletal disease trajectories. We first summarize APOE structure, isoforms, and receptor interactions, then discuss the core mechanistic pathways through which ApoE can shape physiology (inflammation, glucose–insulin responses, mitochondrial/redox homeostasis, senescence, and regulated cell death). Next, we integrate evidence across organ systems to highlight links with adiposity and body composition, bone health, muscle function, and cardiovascular‐metabolic risk, and we conclude by outlining translational opportunities for ApoE genotyping in risk prediction, therapeutic decision‐making, and personalized prevention.

## 2 ApoE Biology and Genetics

ApoE is a multifunctional protein encoded by the human APOE gene. It is found in plasma, cerebrospinal fluid, and lymph, and is produced primarily by hepatocytes in the liver. However, ApoE can also be synthesized in other tissues, including the ovary, immune cells, adipocytes, vascular smooth muscle cells, kidney, adrenal glands, and spleen.

Although ApoE does not cross the blood–brain barrier (BBB), it is also abundantly expressed in the CNS, where it is predominantly synthesized by astrocytes, the major source of ApoE in the brain [4]. It is also produced, to a lesser extent, by activated microglia, vascular mural cells, choroid plexus cells, and in stressed neurons [5, 6, 7, 8, 9, 10].

From the functional point of view, ApoE is a ubiquitous lipid transport protein that binds a variety of lipid species, including cholesterol, phospholipids, and triglycerides (TGs) within lipoprotein particles [11]. It is best known for its central role in lipid transport and lipoprotein metabolism, where lipid distribution and redistribution across tissues and cells is facilitated [12]. However, its functions extend well beyond lipid handling. Inside the cell, ApoE is implicated in a variety of physiological and pathological processes, including the regulation of inflammation and immune function, maintenance of cytoskeletal stability, protection of mitochondrial integrity and function, and promotion of neural tissue repair [13].

The following sections will explore and describe the genetic and biological aspects of ApoE in detail.

### 2.1 Structure, Isoforms, and Evolution

**FIGURE 1:**
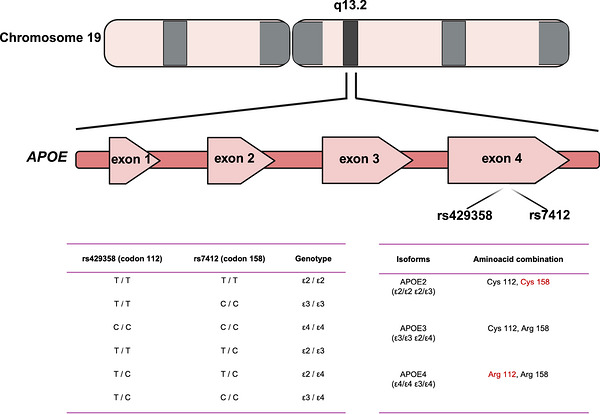
Schematic representation of the human APOE genotype and APOE polymorphisms. (A) The APOE gene is situated in the long arm of chromosome 19 at position q13.3 and is transcribed into 4 exons. The two SNPs, rs429358 and rs7412, are located in exon 4. (B) Two single‐nucleotide polymorphisms (SNPs)—rs429358 at codon 112 and rs7412 at codon 158—define the three major APOE alleles (ε2, ε3, ε4). Specifically, ε2 corresponds to the rs429358 T and rs7412 T combination (TT haplotype), ε3 to T/C, and ε4 to C/C. These alleles give rise to six genotypes: three homozygous (ε2/ε2, ε3/ε3, ε4/ε4) and three heterozygous (ε2/ε3, ε2/ε4, ε3/ε4). (C) Two substitutions at residues 112 and 158 give rise to the three most prevalent ApoE isoforms (ApoE2, ApoE3, and ApoE4). These isoforms differ by the amino acid present at position 112 and/or 158. The residue changes defining ApoE2 (at 158) and ApoE4 (at 112) are highlighted in red. Image created with Biorender.

In humans, the APOE gene is located on the long arm of chromosome 19 (locus 19q13.2) and encodes a 317 amino acid ApoE precursor (NM_000041.4) (Figure 1). The gene consists of four exons and three introns, with a length of 3597 nucleotides [14]. Following cleavage of the 18‐amino‐acid NT signal peptide and subsequent glycosylation, the mature ApoE is secreted as a 299 amino acid polypeptide with an estimated molecular mass of 34.2 kDa.

The human APOE gene contains several single‐nucleotide polymorphisms (SNPs) distributed throughout the sequence. Three major alleles, ε2, ε3, and ε4, are the most common and are thought to have arisen about 7.5 million years ago, after the divergence of humans from other primates [15]. These alleles encode the three major protein isoforms: ApoE2, ApoE3, and ApoE4.

Two nonsynonymous SNPs, 388T>C (rs429358) and 526C>T (rs7412), define the ε2, ε3, and ε4 alleles (Figure 1B) [16]. The combination of these two SNPs gives rise to three principal haplotypes, ɛ2 (388T–526T), ɛ3 (388T–526C), and ɛ4 (388C–526C), and six possible genotypes (ɛ2/ɛ2, ɛ2/ɛ3, ɛ2/ɛ4, ɛ3/ɛ3, ɛ3/ɛ4, ɛ4/ɛ4) [16].

These polymorphisms result in amino acid substitutions at positions 112 and 158 of the mature protein (Figure 1C). Specifically, rs429358 and rs7412 variants comprise cytosine/thymine substitutions that replace arginine (Arg) with cysteine (Cys), thereby resulting in the three common isoforms: ApoE2 (Cys112, Cys158), ApoE3 (Cys112, Arg158), and ApoE4 (Arg112, Arg158) (Figure 1C) [17]. Although the isoforms differ by only one or two residues, these minor variations have a significant impact on the conformation of ApoE and functional properties by altering the interactions between its NT and CT domains. These three isoforms lead to six possible genotypes, homozygous (E4/E4, E3/E3, E2/E2) and heterozygous (E2/E3, E2/E4, E3/E4), each one associated with distinct lipid and disease risk profiles.

ApoE3 is the most prevalent and functionally “neutral” isoform, whereas ApoE2 and ApoE4 differ by a single amino acid substitution that modulates receptor binding and lipid affinity.

Despite APOE expression across mammalian species, this allelic variation is unique to humans. The ε4 is considered ancestral, with the ε2 and ε3 alleles emerging only during human evolution. Comparative sequence analyses indicate that the primary structure of primate ApoE is identical to human ApoE4 [18], but its tertiary structure more closely resembles that of human ApoE3, resulting in receptor‐binding characteristics that are functionally similar to the ε3 isoform [19].

### 2.2 Allelic Frequency and Global Distribution

**FIGURE 2:**
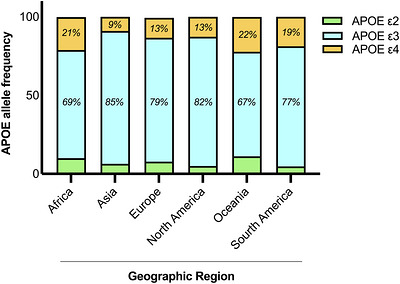
Global distribution of APOE allelic frequencies. Histogram representation of the geographic distribution of the three major APOE alleles (ε2, ε3, and ε4) across human populations, highlighting differences in allele prevalence, with ε3 predominating worldwide and ε4 and ε2 displaying population‐specific enrichment. Image created with Biorender.

The global distribution of the three major APOE alleles varies considerably across human populations (Figure 2). These alleles are thought to have originated approximately 7.5 million years ago, following the divergence of humans from other primates. The *ε4* allele represents the ancestral form, differing from primate ApoE by four amino acid residues. Approximately 150,000–220,000 years ago, further amino acid substitutions gave rise to the *ε3* allele, while the *ε2* variant is believed to have emerged approximately 80,000 years ago through an additional amino acid change  [3, 19].

The estimated timeline of these allelic changes is based on the assumption of neutral evolution [20]. According to the neutral theory of molecular evolution, most genetic variation arises through random drift rather than adaptive selection. Nonetheless, selective pressures on *APOE* alleles have likely been shaped by factors such as age‐related brain function, pathogen resistance (e.g., malaria), climate, fertility, and dietary lipid composition [15].

Among the three alleles, ε3 is the most prevalent, accounting for approximately 80% of all APOE alleles in the global population. The ε2 and ε4 alleles are less common, representing approximately 5–10 and 10–15% of APOE alleles, respectively [21]. Determining APOE allelic frequencies is of paramount importance for understanding their different functional and evolutionary implications.

Geographically, APOE allele frequencies are characterized by a marked variation. The ε3 allele predominates in many regions, accounting for almost 85% in Asia, 69% in Africa, 82% in North America, 77% in South America, and 79% in Europe (Figure 2) [15]. The highest ε4 frequencies are found in Central Africa (40%), Oceania (37%), and Australia (26%), whereas lower ε4 frequencies are observed in Mediterranean and East Asian populations (Figure 2) [22]. In contrast, the ε2 allele shows relatively higher frequency in Africa (9.9%) and Oceania (11.1%) (Figure 2) [15]. These geographic differences likely reflect adaptive responses to distinct environmental pressures and climate conditions.

At the continental scale, ε3 and ε4 allele frequencies exhibit an inverse correlation in Europe, Africa, and North America. In Asian and Oceanian populations, both ε2 and ε4 frequencies tend to rise, when ε3 is less abundant. A strong latitudinal gradient has also been observed: the frequency of ε4 increases with latitude, whereas ε3 decreases (Figure 2). Conversely, ε2 frequencies appear independent of latitude [22].

A more complex pattern has also been observed in which ε4 allele frequencies decline with increasing distance from the equator but rise again at absolute latitudes above 35° [22]. Overall, APOE ε4 is more common among individuals with darker skin pigmentation or those living in regions with low solar irradiance, whereas it is less frequent among populations with moderate melanin levels exposed to high UV radiation. This suggests that the ancestral ε4 genotype may confer an adaptive advantage in low‐UV environments, may be through enhanced vitamin D synthesis efficiency or metabolic resilience.

The emergence of ε3 about 200,000 years ago marked a major shift in APOE evolution even if the precise selective forces and timing of its expansion remain uncertain [20]. Similarly, the appearance of the ε2 allele cannot be dated exactly yet. Because ε2 is absent in population descendent from northern Asian groups that migrated into the Arctic and Americas between 40,000 and 10,000 years ago, it likely arose after these migratory events [20].

### 2.3 Lipid and Cholesterol Metabolism, Physiological Function, and Receptor Interactions

The widespread ApoE tissue production underscores its relevant role in numerous physiological and pathological processes, including lipoprotein metabolism, the transport of fat‐soluble vitamins, regulation of glucose and energy balance, intracellular signaling, vascular growth, tumor dissemination, and brain functions.

Functionally, ApoE plays a pivotal role in the systemic regulation of lipid and cholesterol homeostasis. It coordinates the assembly, transport, and cellular uptake of lipoproteins and participates in receptor‐mediated clearance and intracellular lipid trafficking. ApoE is an essential structural and regulatory component of several circulating lipoproteins, such as chylomicrons, VLDL, intermediate‐density lipoproteins (IDL), low‐density lipoproteins (LDL), high‐density lipoproteins (HDL), and lipoprotein (a) (Lp(a)). Although LDL has often been regarded as largely deficient in ApoE, this assumption has been largely reconsidered. Because the CT ApoE domain mediates lipid binding, it is unlikely that the protein would associate with HDL, VLDL, IDL, and Lp(a) but not with LDL. Indeed, quantitative ultracentrifugation analyses have demonstrated that ApoE constitutes approximately 0.40, 0.10, and 0.38% of the total mass of VLDL, LDL, and HDL, respectively [23]. In fasting plasma, ApoE is distributed mainly among HDL (61 ± 27%), VLDL (35 ± 25%), Lp(a) (4 ± 9%), and LDL (1 ± 1%) [24].

**FIGURE 3:**
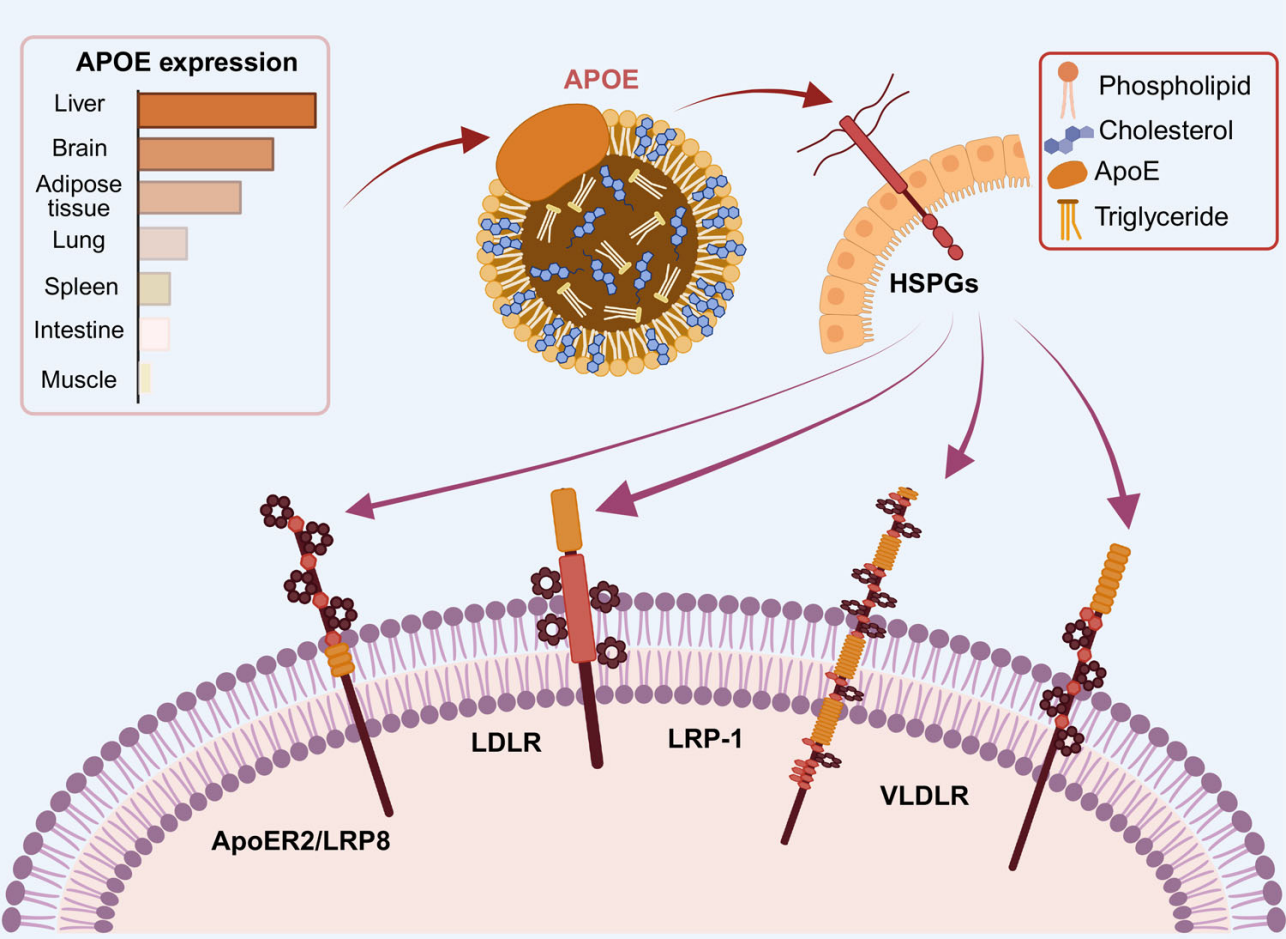
ApoE role in lipoprotein metabolism. ApoE is expressed in various tissues, with higher expression in the liver, brain, and adipose tissue and lower expression in peripheral organs. ApoE serves as a key structural and regulatory component of triglyceride‐ and cholesterol‐rich particles. Acting as a multifunctional ligand, ApoE binds members of the LDL receptor family, including LDLR, LRP1, VLDLR, and ApoER2/LRP8, as well as HSPGs, thereby promoting receptor‐mediated uptake and clearance of lipoprotein remnants. Through these interactions, ApoE contributes to the maintenance of systemic lipid and cholesterol homeostasis and can also modulate cell signaling and extracellular matrix interactions. Image created with Biorender. *Abbreviation*: ApoE: apolipoprotein E; LDLR: low‐density lipoprotein receptor; LRP1: low‐density lipoprotein receptor‐related protein 1; VLDLR: very low‐density lipoprotein receptor; ApoER2/LRP8: low‐density lipoprotein receptor‐related protein 8; HSPGs: heparan sulfate proteoglycans.

Beyond its structural role, ApoE modulates the clearance of circulating lipoproteins and the regulation of plasma lipid levels. By acting as a multifunctional ligand, ApoE binds to a number of cell‐surface receptors, such as the LDL receptor (LDLR), the LDL receptor‐related protein 1 (LRP1), the VLDL receptor (VLDLR), the ApoE receptor‐2/LRP8, and heparan sulfate proteoglycans (HSPG) [25] (Figure 3). Through these interactions, ApoE promotes the hepatic uptake and clearance of TG‐rich lipoprotein remnants, thereby maintaining physiological plasma concentrations of cholesterol and TGs. In addition, its affinity for heparin and HSPG on cell membranes or in the extracellular matrix suggests a potential role in modulating cell signaling and intracellular communication [25].

Dietary lipids absorbed from the intestine are packaged into chylomicrons, which travel through the lymphatic system to enter the bloodstream, delivering TGs and other lipids to peripheral tissues. After energy demands are met and lipid stores replenished within adipocytes and other cells, these chylomicron remnants acquire ApoE, enabling them to bind to members of the LDLR family. Hepatocytes expressing LRP1 subsequently recognize and internalize these ApoE‐enriched remnants, completing their clearance from the circulation [26]. ApoE also contributes to the catabolism of VLDL particles. Once secreted, TG‐rich VLDL particles are hydrolyzed by lipoprotein lipase, and the resulting remnants are cleared via ApoE‐dependent receptor interactions.

Beyond its role in clearing circulating lipoproteins, ApoE is a key regulator of reverse cholesterol transport, the mechanism by which surplus cholesterol is mobilized from peripheral tissues back to the liver for biliary excretion. ApoE enhances this process by associating with HDL particles and stimulating cholesterol efflux through interaction with ATP‐binding cassette (ABC) transporters, particularly ABCA1 and ABCG1 [27]. This activity is essential for preventing the accumulation of cholesterol‐laden macrophages, or foam cells, which drive atherosclerotic lesion formation. In addition, ApoE influences intracellular cholesterol homeostasis by modulating the expression and enzymatic activity of cholesterol‐regulatory proteins and signaling cascades. Through these actions, ApoE affects cellular cholesterol synthesis, uptake, and efflux, thereby maintaining lipid balance at both systemic and cellular levels [25].

ApoE is also produced in adipose tissue where its expression in this compartment is tightly regulated by metabolic and inflammatory cues like peroxisome proliferator‐activated receptor (PPAR)‐γ agonists and tumor necrosis factor (TNF)‐α. Within adipocytes, endogenous ApoE plays a key role in coordinating lipid handling: it affects intracellular TGs and free fatty acid pools, modulates cholesterol synthesis, and regulates genes involved in lipid droplet dynamics and fatty acid oxidation pathways. Notably, ApoE knockout adipocytes exhibit reduced TG accumulation and smaller lipid droplets, whereas reintroducing ApoE restores and augments TG content [28].

It is important to note that a single amino acid change is sufficient to profoundly modify ApoE behavior. The substitutions that distinguish ApoE4 from ApoE3 and ApoE3 from ApoE2 have significant functional consequences. These polymorphic variants differ markedly in structure, despite their subtle sequence differences. This results in isoform‐specific effects on lipid association, receptor affinity, oligomerization tendency, and protein stability. Therefore, understanding the structural distinctions among the isoforms is of paramount importance for interpreting their divergent biological activities.

The critical differences lie at residues 112 and 158, where Cys and Arg are present in different combinations. ApoE3, the most common isoform, contains a Cys and an Arg at positions 112 and 158, respectively. ApoE2 has two Cys (Cys112 and Cys158) and ApoE4 carries two Arg s at these positions. These substitutions reshape the architecture of the receptor‐binding domain and influence lipid interactions [29].

Although ApoE3 and ApoE4 bind to the LDLR with comparable affinity (approximately 20‐fold times greater than apoB100), ApoE2 exhibits markedly impaired receptor binding, retaining only ∼2% of normal activity.

As shown by X‐ray crystallography of the amino‐terminal domain, the presence of Cys‐158 in ApoE2 disrupts the optimal conformation of basic residues in the 136–150 region, a segment essential for LDLR interaction. Structural studies of the NT domain show that, in ApoE3, Arg‐158 forms a stabilizing salt bridge with aspartic acid‐154 (Asp‐154). In ApoE4, this interaction is lost, and Asp‐154 interacts with Arg‐150, altering the entire receptor‐binding region [30].

ApoE structure is highly dynamic, and the isoforms differ in their tendency to form NT/CT domain interactions, generally following the order ApoE4 > ApoE3 > ApoE2. This gradient affects lipid binding strength and thereby influences the biological behavior of each isoform. ApoE4 is characterized by an increased tendency for intramolecular domain interaction. This favors its association with VLDL rather than HDL, reducing its efficiency in HDL‐mediated lipid transport compared with ApoE2 and ApoE3 [31].

## 3 The Core Mechanisms: How ApoE Exerts its Systemic Effects

ApoE has traditionally been framed as a lipid carrier that governs lipoprotein clearance and cholesterol redistribution. However, converging evidence indicates that ApoE functions as a systems‐level regulator that couples lipid handling to inflammatory tone, redox balance, cellular stress responses, and metabolic adaptation across multiple organs. This broader view is essential to interpret the pleiotropic and sometimes discordant associations between APOE genotype and complex phenotypes, including neurodegeneration, cardiometabolic disease, and age‐related functional decline, where the same isoform may confer risk or protection depending on tissue context, lipidation state, sex, age, and environmental exposures.

The present section delineates a set of mechanistic axes through which ApoE is proposed to exert systemic effects. Rather than treating neurobiology and peripheral physiology as separate domains, emphasis is placed on shared pathways (immune signaling, oxidative‐mitochondrial homeostasis, glucose–insulin regulation, and cell‐fate programs) that operate in both the brain and the cardiovascular‐metabolic compartment. Organ‐specific manifestations are discussed where appropriate, but the guiding principle is that ApoE‐dependent modulation of these fundamental processes provides a unifying explanation for multiorgan vulnerability and heterogeneity across studies.

Accordingly, four interlinked mechanisms are examined: (i) inflammation as a bidirectional interface between lipid metabolism and tissue injury; (ii) oxidative stress and mitochondrial function as determinants of energetic capacity and resilience; (iii) insulin signaling and glucose homeostasis as early modulators of neural and systemic metabolism; and (iv) senescence and regulated cell death as endpoints through which chronic stress is translated into dysfunction and, ultimately, disease progression.

### 3.1 Inflammation in Brain and in Cardiovascular System

Inflammation plays a pivotal role in the progression and severity of several neurodegenerative diseases (NDs) by facilitating or exacerbating the formation of both amyloid β (Aβ) and neurofibrillary tangles  [32, 33]. Beyond its classical role in lipid biology, ApoE has emerged as a key modulator of neuroinflammation [34]. Isoform‐specific differences are particularly evident at the BBB level: in the presence of ApoE4, pericytes activate a cyclophilin A–MMP9 pathway, which compromises BBB integrity, whereas ApoE3 and ApoE2 maintain barrier stability [35]. Genome‐wide association studies (GWASs) further reinforce the link between neuroinflammation and the development of AD [36, 37, 38], identifying risk variants in immune‐related genes such as complement receptor‐1, CD33, and triggering receptor expressed on myeloid cells‐2 (TREM2) [39, 40, 41]. As a ligand for TREM2, ApoE may regulate TREM2‐dependent inflammatory signaling [42, 43, 44].

It was shown in primary glial cultures that inflammation modulates ApoE expression and secretion in an isoform‐dependent manner [45]. More specifically, ApoE4 drives more pronounced proinflammatory responses than ApoE3 or ApoE2 (including higher production of nitric oxide—NO), TNF‐α, IL‐6, IL‐1β, and IFNγ [46, 47, 48]. In a mouse model with inducible astrocytic expression of human APOE isoforms, astrocyte‐derived ApoE3 was shown to suppress IL‐6 and IL‐1β, whereas ApoE4 increases all major proinflammatory cytokines [49]. Elevated baseline TNFα levels have been observed in microglia expressing the APOE4 variant, suggesting that this isoform contributes to heightened inflammatory responses in primary immune cells. ApoE also influences the inflammatory activity of macrophages in peripheral tissues in an isoform‐dependent fashion. ApoE4 is linked to morphological changes and increased cytokine secretion [47, 50].

Despite these proinflammatory tendencies, ApoE can also exert anti‐inflammatory actions. Through ApoER2 and VLDLR, ApoE attenuates Toll‐like receptor (TLR)‐dependent signaling and reduces cytokine production [51]. Conversely, inflammatory signaling can downregulate ApoE levels in astrocytes, suggesting the presence of a regulatory feedback loop [52]. Anti‐inflammatory properties have also been demonstrated for recombinant ApoE3 and ApoE‐mimetic peptides, which suppress glial activation in models of lipopolysaccharide (LPS) exposure or injury‐induced inflammation [53, 54, 55, 56]. Moreover, ApoE can limit complement‐mediated inflammation by binding C1q and dampening activation of the classical complement cascade [57].

These isoform‐specific differences are reproduced in vivo using mice humanized for APOE. Animals expressing ApoE4 display stronger inflammatory responses to LPS and Aβ than those expressing ApoE3 or ApoE2 [58]. In APP transgenic mice, ApoE4 enhances Aβ deposition, microgliosis, and astrocyte dysfunction, whereas ApoE2 shows protective effects [59]. Of note, lipidation status influences these outcomes: only lipid‐bound ApoE4, not its lipid‐poor form, exacerbates Aβ‐triggered inflammation, while both ApoE2 and lipidated ApoE3 generally attenuate the response [54]. Variability in these responses may stem from differences in the formation of Aβ aggregates or may be influenced by cholesterol metabolism and HDL levels, particularly in ApoE4 carriers, which are known to modulate neuroinflammatory pathways [60].

Given the differing results reported in the literature, it is natural to wonder why ApoE has different both anti‐inflammatory and proinflammatory properties. This contrasting effects of ApoE is likely influenced by its genetic variants. Indeed, ApoE4 strongly promotes NF‐κB activation and proinflammatory gene expression, whereas ApoE2 induces a more anti‐inflammatory phenotype in glial cells [61, 62].

Hyperinflammation also contributes to the development and progression of atherosclerosis. This chronic condition is driven by elevated blood lipids and persistent vascular inflammation. In the early stages, monocyte‐derived macrophages play a significant role by infiltrating the vessel wall and forming foam cells after internalizing oxidized ApoB‐containing lipoproteins [63, 64]. This inflammatory environment attracts further innate and adaptive immune cells, including neutrophils, natural killer (NK) cells, NK T cells, T cells, and B cells [65]. ApoE, whether transported via circulating lipoproteins or produced locally within the vessel wall and by macrophages, plays a protective role throughout these stages by regulating lipid balance and inflammatory signaling. Indeed, atherosclerosis progresses more aggressively in ApoE‐deficient mice (ApoE^−/−^) due to excessive lipid accumulation and intensified vascular inflammation linked to defective lipoprotein metabolism [66].

It has also been observed that extracellular ApoE plays an anti‐inflammatory role in macrophages by suppressing the production of inflammatory cytokines. This occurs through the inhibition of TLR pathways, specifically TLRs 3 and 4, by blocking c‐Jun and JNK signaling. These effects depend on distinct receptor interactions: binding to HSPGs inhibits TLR3 signaling, while interaction with LRPs suppresses TLR4 activation [67]. Macrophage‐derived ApoE plays a key role in modulating inflammation by enhancing cholesterol efflux through several mechanisms, for instance through interaction with the transporter ABCA1 [68], whose expression is upregulated by activation of the PI3K–PKCε–Sp1 signaling pathway [69]. Overall, this regulation reduces intracellular cholesterol build‐up and prevents cholesterol‐induced activation of the inflammasome [70]. Endogenously produced ApoE is particularly effective in facilitating this process, potentially more so than externally supplied lipid‐free ApoE in vitro [63, 64, 71]. This efficiency is likely due to the fact that intracellularly produced ApoE is secreted along with cholesterol and phospholipids, forming an ApoE–lipid complex that interacts with the ABCA1 transporter to drive further cholesterol efflux [71]. It has been shown that ApoE produced within macrophages is essential for optimal cholesterol removal and reverse cholesterol transport, whereas circulating ApoE alone may be insufficient [64, 65]. ApoE also contributes to anti‐inflammatory protection by increasing microRNA‐146a, which targets TRAF6 and IRAK1 to suppress NF‐κB signaling [72].

ApoE isoforms differentially affect oxidative stress. Although purified ApoE4 protein was shown to enhance LDL oxidation more than ApoE2 or ApoE3 in vitro, macrophage studies have shown that cells expressing ApoE2 may promote LDL oxidation more strongly, likely because lower secretion of ApoE2 limits its extracellular antioxidant capacity. Despite this nuance, macrophages expressing ApoE4 generally display increased oxidative stress, including elevated ROS levels, membrane oxidation, and NO production, compared with ApoE3‐expressing cells [16, 50, 69].

### 3.2 Oxidative Stress and Mitochondrial Function

Over the years, it has become evident that ApoE influences biological processes far beyond its well‐established function in lipid handling. Studies in ApoE‐deficient mice demonstrate that the absence of this protein produces marked vascular stress, with pronounced oxidative damage in the aorta and broad transcriptional alterations involving redox balance, inflammatory pathways, and endothelial regulation [72]. A more systemic view emerged from work using d‐galactose‐induced ageing models, where ApoE KO impaired cognition through mechanisms that involved both oxidative stress and disorders along the gut–brain axis [67]. ApoE itself is vulnerable to oxidative modification. Molecular analyses have shown that 4‐hydroxynonenal (HNE), a product of lipid peroxidation, can covalently bind the Cys residues present in ApoE2 and ApoE3, thereby reducing free HNE accumulation and limiting its ability to damage other proteins. ApoE4, which lacks these Cys, cannot form such adducts and is therefore less effective at neutralizing reactive aldehydes [68].

There is now growing evidence linking the ApoE genotype to oxidative stress in the brain. ApoE4 has repeatedly been associated with higher ROS production, increased protein and lipid oxidation, and reduced antioxidant defenses. These effects have been observed across multiple experimental settings, including synaptosomal preparations, mouse models, and clinical samples where ApoE4 carriers display elevated oxidative biomarkers and lower cerebral oxygen utilization [69, 70, 73]. Transcriptomic studies have implicated pathways such as Notch signaling in these processes, and ApoE4 has been linked to diminished expression of key antioxidant and redox‐related proteins, including SOD2, PRDX5, HSPD1, as well as reduced glutathione capacity and greater levels of lipid peroxidation products such as malondialdehyde [74, 75]. Additional mechanisms include activation of eicosanoid and lipoxygenase pathways, which further fuel oxidative stress in ApoE4‐expressing brains [76].

Mitochondrial function is another major point of divergence among the ApoE isoforms. Experiments in hepatocyte lines, primary astrocytes, neurons, and ApoE‐targeted replacement mice have consistently shown that cells expressing ApoE3 maintain more favorable energetic profiles than those expressing ApoE4, generally displaying higher ATP availability and more robust respiratory activity [77, 78, 79, 80]. Reduced levels of PGC‐1α, SIRT3, and other regulators of mitochondrial biogenesis and dynamics have been reported in cells and individuals carrying ApoE4. Moreover, cells and individuals carrying ApoE4 also exhibit lower expression of mitofusin‐1, mitofusin‐2, and DRP1. This suggests an impaired capacity to sustain healthy mitochondrial networks [81, 82]. Some studies have reported no significant differences in total ATP levels between isoforms but have noted shifts in ATP origin. ApoE4‐expressing endothelial cells, for example, rely more on oxidative phosphorylation and less on glycolysis, which may limit metabolic flexibility under stress [83, 84]. ApoE4 has also been implicated in defective autophagy and mitophagy, in part through repression of the FoxO3a transcription factor [85].

Proteolytic processing can result in isoform‐specific differences. In neurons, ApoE4 is more prone to cleavage, producing fragments that contain both lipid‐ and receptor‐binding regions. These fragments compromise mitochondrial function and promote neurotoxicity, and their presence correlates with reduced expression of respiratory complexes I, IV, and V in ApoE4 models [86]. Consistent reductions in components of the oxidative phosphorylation system have been documented in ApoE4‐expressing cells, targeted replacement mice, and postmortem human brain tissue, including decreased cytochrome oxidase activity and diminished levels of ATP synthase subunits [79, 84, 87, 88]. Outside the brain, however, the impact of ApoE isoforms on mitochondrial respiration remains less clear, with only a small number of studies reporting minimal differences between ApoE3 and ApoE4 [89].

ApoE4 may also alter mitochondrial dynamics. Some reports have described greater mitochondrial fusion in ApoE4‐expressing hippocampal tissue and astrocytes, whereas others observed reduced levels of both fusion and fission proteins in ApoE4 carriers, pointing to an overall decline in mitochondrial plasticity [78, 82, 84, 90]. ApoE4‐derived fragments add further complexity by promoting mitochondrial fragmentation and increasing fission activity in neuronal and hippocampal tissue [91].

Taken together, current evidence indicates that ApoE plays a broad role in maintaining redox and mitochondrial homeostasis. ApoE4 consistently shows reduced antioxidant capacity, altered lipid‐derived reactive species handling, impaired mitochondrial function, and disrupted organelle dynamics. Although many mechanisms have been described in neural tissue, it remains uncertain to what extent these isoform‐specific effects extend to other organs. Understanding how ApoE variants influence mitochondrial biology across different systems is crucial for clarifying their contribution to human health and disease.

### 3.3 Insulin Signaling and Glucose Homeostasis

Most studies carried out over the last few years have investigated the association between ApoE and altered glucose metabolism in the brain. Disruptions in cerebral glucose metabolism represent one of the earliest abnormalities observed in AD [92], and this deficit tends to be more pronounced in individuals carrying the ApoE4 allele [93, 94]. Although the exact mechanisms by which different ApoE isoforms influence glucose metabolism remain unclear, several studies in humans and mouse models suggest that ApoE isoforms influence both glucose uptake and downstream metabolic processing [95, 96, 97]. For example, reduced glucose metabolism and insulin signaling were found to correlate more strongly with ApoE4 status. This was seen in cognitively healthy older adults. Transgenic mice expressing human ApoE isoforms demonstrated that ApoE2, not ApoE4, enhances brain glucose metabolism [98, 99].

Astrocytes, which support neuronal energy needs, also show isoform‐dependent metabolic behavior. Human astrocytes expressing ApoE4 preferentially divert glucose toward glycolysis while displaying reduced mitochondrial respiration [100]. Similar metabolic alterations, together with disrupted lipid homeostasis and lysosomal dysfunction, have been reported in several neuronal systems exposed to ApoE4 [79, 95, 101]. Overall, it appears that glycolysis is more pronounced in the presence of ApoE2, whereas ApoE4 is linked to an increased conversion of glucose to lactate. In line with this metabolic shift, individuals carrying an ε4 allele consistently show reduced cerebral glucose metabolism on imaging studies [95, 98]. Mechanistically, ApoE4 promotes a rerouting of glucose into alternative pathways, including the pentose phosphate pathway (PPP) and the tricarboxylic acid cycle, which favors biosynthesis at the expense of efficient energy production [102]. This metabolic pattern results in lower glucose oxidation, reduced oxygen consumption, and diminished whole‐body energy expenditure, mirroring a “Warburg‐like” metabolic profile that has been observed in ApoE4 astrocytes, brain tissue, and even systemically in ApoE4 mice well before overt cognitive symptoms appear [101, 103, 104]. In support of these findings, ApoE4‐expressing mice show reduced levels of GLUT3, the main neuronal glucose transporter, compared with ApoE3 carriers [95].

ApoE4 also intersects with insulin‐related pathways. Altered insulin signaling has been well documented in both AD brain tissue and experimental models [105, 106], and a recent work indicates that ApoE4 contributes directly to this dysregulation in an age‐dependent manner [107, 108]. ApoE4 can interfere with insulin receptor function by binding to the receptor and hindering insulin from interacting with its normal target. In addition, ApoE4 tends to self‐aggregate more readily than other isoforms, and these aggregates may physically obstruct the receptor–ligand interaction. As a result, insulin‐driven pathways, including glycolysis and mitochondrial respiration, are dampened in ApoE4‐expressing cells [107]. Beyond the brain, individuals with the ε4 allele also display changes in signaling pathways connected to insulin and metabolic regulation, including PPARγ and its co‐activator PGC‐1α; increasing PGC‐1α expression experimentally has been shown to counteract some of the metabolic deficits linked to ApoE4 [92, 95].

Importantly, ApoE‐related effects are not confined to the brain. Several studies point toward a broader role for ApoE in energy regulation, insulin sensitivity, and adipose tissue function [109, 110]. In ApoE^−/−^ mice, hyperuricemia exacerbates aortic plaque load and cell apoptosis, which is associated with activation of the NLRP3 inflammasome [111, 112]. In obesity, ApoE expression in adipose tissue is downregulated and mice lacking ApoE display improved glucose tolerance and insulin sensitivity under a high‑fat diet. Mechanistically, ApoE seems to influence the NLRP3 inflammasome in adipose tissue, which links local inflammatory signaling to systemic glucose and insulin dysregulation [113]. Human studies reflect this complexity: in older adults, ApoE status correlates with differences in glucose tolerance, with sex‐specific effects: women carrying the ε4 allele show higher OGTT glucose values, whereas men show the opposite trend despite similar insulin levels [114]. These findings suggest that ApoE isoforms influence the way glucose is used by the body's tissues in a way that is affected by age, sex, and metabolic state.

Accumulating data also connect ApoE to type 2 diabetes (T2DM). Several meta‐analyses and clinical studies indicate that both ApoE genotype and circulating ApoE levels correlate with diabetes risk and its complications. For instance, ApoE4 carriers tend to display poorer glucose clearance and altered insulin/leptin responses even under modest dietary challenge [115]. A meta‐analysis of 59 case–control studies reported that ε4 and genotypes such as ε2/ε2, ε3/ε4, and ε4/ε4 were associated with increased T2DM risk [116]. Higher circulating ApoE concentrations have also been linked to future diabetes onset in individuals with prediabetes [117], and isotope‐based kinetic work shows increased ApoE production in both VLDL and HDL fractions in people with obesity‐associated T2DM [118]. However, findings across studies are not entirely consistent: while some analyses identify ε4 as the primary risk allele [119], others report that ε2 modestly increases susceptibility to T2DM [120].

ApoE genotype also appears to influence the risk of diabetes complications. Specific ApoE variants (ε2/ε2, ε2/ε3, ε2/ε4) have been associated with diabetic kidney disease [121], and ε3/ε4 or ε4 allele alone correlates with increased cardiovascular complications among diabetic individuals [122]. Similarly, a higher incidence of diabetic peripheral neuropathy has been reported in ApoE4 carriers [123].

Taken together, the available evidence indicates that ApoE plays a wide‐ranging role in metabolic regulation. Its effects vary by isoform, sex, and metabolic context, influencing glucose utilization in the brain, insulin signaling, adipose tissue inflammation, and systemic metabolic homeostasis. While ApoE is not a direct cause of diabetes, certain ApoE genotypes may heighten susceptibility to impaired glucose metabolism and increase vulnerability to diabetes‐related complications. These insights suggest that ApoE genotype may eventually support more personalized strategies for identifying individuals at greater metabolic risk and tailoring preventive or therapeutic interventions accordingly.

### 3.4 Cellular Senescence, Apoptosis, and Longevity

When subjected to persistent stress, cells can enter a stable state known as cellular senescence [124]. In this state, cell division is permanently arrested, and the cell adopts a distinct metabolic and secretory profile [125, 126, 127]. Senescent cells release a mixture of cytokines, chemokines, proteases, and growth factors, collectively termed the senescence‐associated secretory phenotype (SASP), that can disrupt tissue structure and amplify inflammation [128, 129, 130].

Although it remains uncertain whether senescence drives neurodegeneration or arises as a consequence of it [131], features of senescence have been detected in neurons, astrocytes, microglia, endothelial cells, and oligodendrocyte precursor cells in both AD models and human AD brain tissue [132, 133, 134, 135].

Because ApoE is genetically linked to AD, its relationship to ageing and senescence has become a topic of growing interest. Several studies suggest that ApoE4 promotes a prosenescent intracellular environment by diverting cholesterol to lysosomes, triggering lysosomal stress, aberrant mTORC1 activation, and a heightened SASP response  [136, 137, 138]. ABCA1 appears to contribute to this process by delivering cholesterol into lysosomes, which may worsen lysosomal dysfunction [136]. Evidence from human mesenchymal progenitor cells indicates that ApoE accumulation itself can be a driver of senescence: aged progenitor cells show increased ApoE levels, while loss of ApoE confers resistance to senescence. Mechanistically, ApoE destabilizes nuclear architecture by promoting the degradation of lamina components and the heterochromatin‐associated protein KAP1 through the autophagy–lysosomal system, ultimately weakening heterochromatin integrity and triggering senescence [139].

Work in humanized ApoE target‐replacement mice supports these findings. Aged ApoE4 mice accumulate senescent neurons within the hippocampus and show activation of mTOR and endosome–lysosome–autophagy pathways. These changes coincide with disrupted energy metabolism, including reduced citrate synthase activity, diminished ATP content, and notably low acetyl‐CoA levels, suggesting that energy deficits contribute to the senescence‐prone phenotype of ApoE4 neurons [140]. Additional evidence comes from doxorubicin‐induced senescence models, where ApoE4 knock‐in mice displayed a stronger senescent response than ApoE3 mice, pointing to a genotype‐dependent vulnerability to stress‐induced ageing processes [141]. In human postmortem tissue, ApoE4 carriers also show more extensive senescence signatures across neuronal populations, accompanied by oxysterol accumulation, ABCA1‐related lysosomal abnormalities, and mTORC1 activation [138]. Interestingly, ApoE‐mediated senescence is not confined to neural tissue: in prostate cancer models, tumor‐derived ApoE can bind TREM2 on neutrophils, pushing them into a senescent‐like state and supporting tumor progression [142].

ApoE also affects pathways governing apoptosis, and ApoE4 is particularly implicated in enhancing cell death under stressful conditions. Early studies showed that ApoE4 can promote apoptosis in neuronal cell lines through mechanisms involving LRP‐dependent signaling [143]. In contrast, in pancreatic cancer cells, ApoE2 activates ERK1/2‐CREB signaling to increase BCL‐2 stability, maintain mitochondrial membrane potential, prevent cytochrome *c* release, and ultimately protect against apoptosis [144]. ApoE displays antiapoptotic effects in certain immune contexts as well; for example, its interaction with regulatory T cells suppresses caspase‐mediated apoptosis and enhances cell survival [145]. However, the data reported in the literature remain inconsistent. In chronic lymphocytic leukemic cells, ApoE induces ferroptosis rather than apoptosis by promoting lipid peroxide accumulation [146]. Conversely, other reports indicate that ApoE can activate PI3K/AKT signaling, inhibit ferritin autophagic degradation, and thereby reduce iron‐driven lipid peroxidation [147]. Lipoprotein‐associated ApoE4 appears less effective at preventing apoptosis than other isoforms, and ApoE4‐containing VLDL can blunt the antiapoptotic effects of HDL through interactions with LDL‐family receptors [148]. Moreover, ApoE4 increases neuronal susceptibility to Aβ‐induced lysosomal membrane destabilization and apoptosis, likely by forming reactive intermediates capable of inserting into lysosomal membranes [149].

Given the strong genetic links between ApoE and AD, its relevance to human longevity has been examined extensively. Many population studies report that the ε4 allele is under‐represented among individuals who reach advanced age, suggesting a detrimental effect on lifespan [150, 151, 152, 153, 154, 155, 156]. By contrast, ε2 is more commonly found in long‐lived individuals [157], and metabolomic profiling of ApoE2 carriers has revealed a distinct lipid and metabolite signature that may contribute to healthier ageing trajectories [158]. Interestingly, several cohorts have shown an enrichment of the ε3/ε3 genotype among older adults and their descendants, implying that ε3 may offer a modest survival benefit compared with ε2/ε3 or ε3/ε4 genotypes [159]. Experimental evidence from tau transgenic mice also supports a survival advantage for ApoE3 over ApoE4 in models of tauopathy [160], consistent with the broader view that ApoE4 tends to confer a toxic gain‐of‐function in neurodegeneration, whereas loss of ApoE is relatively protective [161]. The influence of ApoE on longevity also appears to intersect with comorbid conditions: for instance, diabetes shortens lifespan in ε3 and ε2 carriers, indicating that metabolic context modifies genotype‐related effects [162], while ApoE2 has been associated with extended lifespan independent of AD risk, partly linked to favorable lipid profiles and preserved physical function [163].

Although mechanistic understanding is still evolving, the collective evidence shows that ApoE affects ageing on multiple levels: by shaping lipid metabolism, mitochondrial function, inflammatory tone, and the balance between senescence and apoptosis. Clarifying how specific ApoE isoforms modulate these fundamental processes may open new avenues for delaying age‐related decline, preventing NDs, and improving metabolic resilience across tissues.

## 4 ApoE and the Spectrum of Pathophysiological Conditions

**FIGURE 4:**
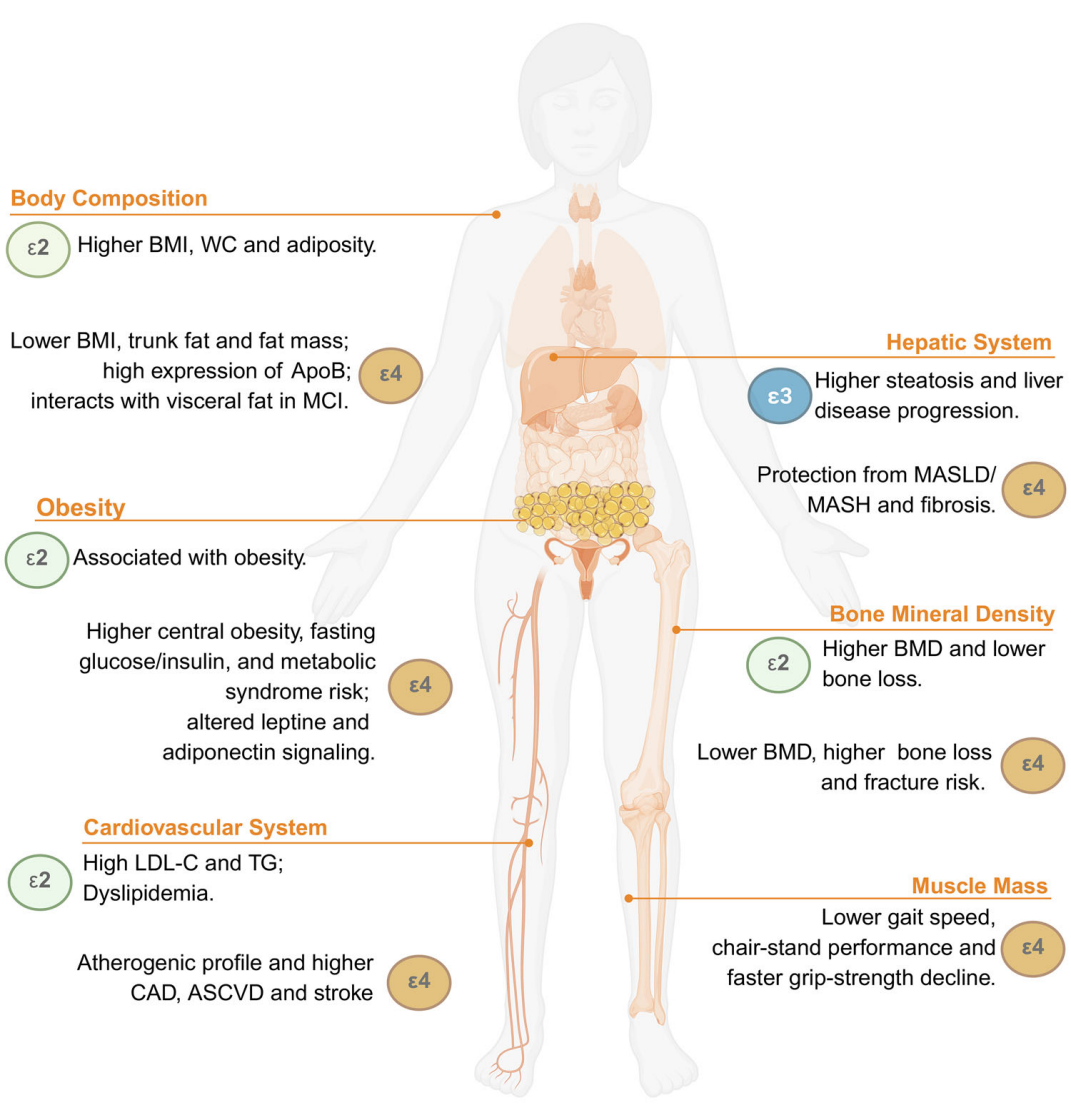
APOE genotype across pathophysiological conditions. Schematic overview summarizing the associations between APOE ε2, ε3, and ε4 alleles and multiple systemic conditions. ε2 is associated with an increased risk of adiposity, dyslipidemia, and obesity, but also with higher bone mineral density and reduced bone loss. In contrast, ε4 is commonly associated with lower total body mass but greater central adiposity, adverse cardiometabolic profiles, impaired glucose and insulin regulation, reduced bone mineral density, increased fracture risk, and a more rapid decline in muscle performance. In the liver, ε3 has been associated with increased steatosis and disease progression, whereas ε4 appears to confer relative protection against MASLD/MASH and fibrosis. Image created with Biorender.

The three major APOE isoforms show distinct, context‐dependent associations with disease risk and underlying pathophysiology (described in the following sub‐paragraphs), contributing to heterogeneous and occasionally discordant findings across studies. To support an integrated interpretation of this complexity, Table 1 and Figure 4 summarize and compare the isoform‐specific contributions of APOE ε2, ε3, and ε4 to disease onset and progression, highlighting both shared and opposing effects.

**TABLE 1:** Isoform‐specific effects of APOE ε2, ε3, and ε4 on modulating disease susceptibility.

		ApoE isoforms		
	Disease/condition	ε2	ε3	ε4
Body composition	BMI (adults)	Increased [152, 164] Protective [165]	Neutral	Protective [152] Increased [165, 166]
	Waist circumference (adults)	Increased [164]	Neutral	No data
	BMI (children)	No data	Neutral	Protective [167]
	Waist circumference (children)	No data	Neutral	Protective [167]
	Body weight (older adults with MCI or AD)	No data	Neutral	Protective, lower adiposis only women [171]
	BMI (cognitively healthy older)	No data	Neutral	Protective [172]
Obesity/altered insulin signaling	Glucose metabolism and altered insulin signaling (cognitively healthy older adults)	No data	No data	Increased [79, 95, 98, 99, 100, 101, 105, 106, 107, 108]
	Obesity	Increased [184, 185]	No data	Increased [174, 175, 176] Increased in women [178] Protective [166, 177]
	Glucose/insulin levels and insulin resistance	No data	No data	Increased [183]
	Type 2 diabetes mellitus	Increased [120]	No data	Increased [116, 119]
Bone mineral density	Low BMD, bone loss, fracture risk	Neutral [205, 206, 207] Protective [211]	Neutral	Increased [188, 189, 190, 194] [199, 200, 201, 202] Increased hip fracture incidence [191] Higher % previous fracture [195, 196] Lower hip BMD [190, 197] Reduced lumbar spine BMD [192, 193, 198] Neutral [203, 204, 205, 206, 207, 208, 209, 210]
Muscle mass	Slower gait speed, poorer performance on chair‐stand tests, greater decline in handgrip strength, higher risk of disability	No clear protective or risk associations	Neutral	Increased [219, 220, 221]
Cardiovascular system	Atherosclerosis, dyslipidemia, coronary artery, CVD	Protective [223, 224, 229, 230] Increased [231, 232, 233]	Neutral	Increased [223, 224, 225, 226, 227, 228, 243]
	Atherosclerotic cardiovascular disease	No data	No data	Increased [241]
	Coronary lesions	No data	Increased [237]	Increased [237, 242]
	Myocardial infarction	Neutral [238] Protective [239, 240]	Neutral [238, 239] Increased [240]	Increased [238] Neutral [239] Increased [240]
Hepatic system	MASH, MASLD, HCC, cirrhosis	Neutral	Increased [251, 260]	Protective [252, 253, 260]

Abbreviations: BMI, body mass index; MCI, mild cognitive impairment; AD, Alzheimer's disease; BMD, bone mineral density; CVD, cardiovascular disease; MASH, metabolic dysfunction‐associated steatohepatitis; MASLD, metabolic dysfunction‐associated steatotic liver disease; HCC, hepatocellular carcinoma.

### 4.1 Body Composition

The influence of the APOE genotype on body composition has been examined in animal models and, to a lesser extent, in human populations. However, existing findings remain conflicting. Several studies report that the ε2 allele is associated with greater adiposity. In adults between 20 and 85 years of age and in middle‐aged Caucasian men, ε2 carriers showed higher waist circumference and BMI [164]. A pooled analysis of seven longitudinal cohorts similarly linked ε2 with elevated BMI, whereas ε4 was associated with lower BMI, particularly among older Caucasian individuals [152]. By contrast, data from the ARIC study suggested the opposite pattern: ε4 correlated with higher BMI, and ε2 with lower BMI [165]. In a smaller case–control cohort, ε4 was positively associated with BMI, but only in overweight or obese subjects (BMI > 25 kg/m^2^) [166]. These conflicting results underscore the strong influence of age, ethnicity, and metabolic background on genotype–phenotype relationships.

In children, the pattern seems different. An investigation in 8‐year‐olds found that ε4 carriers had lower BMI, smaller waist circumference, and reduced trunk fat compared with non‐ε4 children [167], suggesting that APOE‐related effects on body composition may shift across the lifespan. Animal models add another layer of complexity. Mice expressing apoE4 showed greater increases in adiponectin when exposed to an obesogenic diet compared with apoE3 mice, although the mechanisms linking apoE4 to lean mass or adipokine biology remain poorly understood [168].

Additional insights come from work on normal‐weight obesity syndrome, a phenotype characterized by normal BMI but elevated fat mass. In these subjects, APOE genotype influenced lipid traits: ε2 was linked to higher apolipoprotein A1 concentrations and a substantially reduced likelihood of dyslipidemia, whereas ε4 was associated with elevated apolipoprotein B levels [169].

Increasing evidence indicates that metabolic health, diet, body composition, and physical activity interact with APOE genotype to influence cognitive outcomes. In patients diagnosed with mild cognitive impairment (MCI), the presence of the ε4 allele was found to modify the relationship between body fat distribution, particularly visceral fat, and waist‐to‐hip ratio, and cognitive performance [170]. In older adults with MCI or early to moderate AD, ε4 was linked to lower body weight and reduced fat mass in women, but not in men [171]. More recently, cognitively healthy older adults carrying ε4 also exhibited lower BMI and lower overall body fat percentage than noncarriers [172].

Taken together, the available studies point toward a complex, context‐dependent relationship between APOE genotype and body composition, shaped by age, sex, metabolic status, and environmental factors. Although intriguing patterns have begun to emerge, robust longitudinal human studies with prospective genotyping are still needed to clarify whether these associations reflect causal mechanisms or population‐specific observations.

### 4.2 Obesity

Obesity is a multifactorial condition shaped by complex interactions among genetic background, epigenetic modifications, environmental exposures, and lifestyle patterns. Although numerous genetic loci have been implicated, the regulatory pathways linking specific genotypes to adiposity remain only partially understood. A recently published work has begun to clarify how ApoE contributes to adipose tissue biology. In 2024, Jiang and colleagues demonstrated that adipocyte‐derived ApoE influences the browning of white adipose tissue, the formation of beige adipocytes, and thermogenic capacity, thereby affecting whole‐body energy balance [173]. Several population studies have examined whether the APOE ε4 allele increases susceptibility to obesity. Associations have been reported in Brazilian [174], Saudi Arabian [175], and Egyptian [176] cohorts, whereas other studies have linked ε4 to lower BMI measurements [166, 177], illustrating substantial heterogeneity across populations. In a case–control study, Alharbi et al. observed a higher prevalence of ε4 among individuals with obesity [166], and another study reported an increased risk of central adiposity in ε4 carriers [177]. Findings from the Rancho Bernardo Study also indicated that ε4 was associated with obesity independently of dyslipidemia in women with a family history of diabetes [178]. Moreover, the ε4 allele was more frequent among individuals with metabolic syndrome, irrespective of sex [179]. Among obese men, ε4 genotype has been linked to increased glucose and insulin concentrations [180].

Several mechanisms that may underlie the association between obesity and ApoE4 have been suggested. For instance, APOE isoforms have been shown to differentially interact with hormones important in nutrient sensing and homeostasis, including adiponectin [181] and leptin [182], both of which play central roles in nutrient sensing and energy homeostasis. ApoE also modulates appetite at the level of the hypothalamus [183], although potential isoform‐specific effects have not yet been defined. In metabolic studies, ε4 has been associated with higher fasting glucose and insulin levels, a younger age of metabolic syndrome onset, and unfavorable lipid and glucose profiles in obese men, consistent with an increased tendency toward insulin resistance [184]. Despite this, some reports suggest that the ε2 allele, rather than ε4, may be associated with greater obesity risk in certain populations [185, 186].

Taken together, the available findings highlight a complex and sometimes contradictory relationship between APOE genotype and obesity. Differences across ethnic groups, study designs, and environmental exposures likely contribute to the inconsistencies. Given this variability, approaches based on precision medicine or personalized nutrition have been proposed to tailor metabolic interventions, particularly for individuals carrying risk‐associated APOE alleles such as ε4.

### 4.3 Bone Mineral Density

Bone tissue is constantly remodeled: osteoblasts build new bone tissue, while osteoclasts break down old or damaged tissue. When this balance shifts, however, bone density begins to fall, which can eventually increase the risk of osteoporosis. As osteoblasts and adipocytes originate from the same pool of mesenchymal stem cells, changes in lipid metabolism can easily affect skeletal biology. This connection becomes evident in studies of ApoE‐deficient mice, where the absence of ApoE disrupts normal stem cell differentiation. These animals show less bone formation and accumulate more fat within the marrow space, although the molecular details behind these changes are still not fully understood [187].

Several hypotheses have been proposed to explain how APOE polymorphisms may contribute to variations in bone mineral density (BMD). One possibility is that ApoE4, which has higher affinity for certain lipoprotein receptors, lowers circulating vitamin K levels, an essential cofactor for osteocalcin maturation, thereby impairing bone formation [188]. Another proposed mechanism links ApoE4‐related dyslipidemia to bone biology: elevated LDL and accumulation of oxidized lipids in the bone matrix may inhibit osteoblast differentiation and favor bone loss [189].

Since the early 2000s, numerous observational studies have associated the ε4 allele with adverse skeletal outcomes. Reported findings include lower hip BMD [190], increased hip fracture incidence [191], reduced lumbar spine BMD in postmenopausal women [192] and lower BMD at both the lumbar spine and femoral neck [193]. ε4 has also been linked to unfavorable bone microarchitecture [194], a higher prevalence of previous fractures [195], and a twofold increase in self‐reported hip fractures [196]. Additional studies have corroborated elevated risks of hip and wrist fractures in ε4 carriers [197] as well as greater age‐related loss of spinal BMD [198]. Large cohort analyses have likewise supported associations between ε4 and lower BMD at specific skeletal sites, including the lumbar spine, greater trochanter, and hip [190, 199, 200, 201]. In postmenopausal Brazilian women, ε4 carriage was associated with reduced bone formation and increased susceptibility to osteoporosis and fractures [202].

However, the evidence is far from consistent. Multiple studies, including the large Rotterdam cohort of more than 5000 participants, reported no significant relationship between ε4 and BMD, bone loss, or fracture risk [203, 204]. In addition, three GWASs failed to identify APOE variants associated with BMD [205, 206, 207], and several population‐based analyses detected no links between ε4 and BMD at any skeletal site or hip fracture incidence [208, 209, 210].

There is also interest in whether ApoE2 might exert protective effects on bone. In a longitudinal study of more than 2600 women, carriers of the ε2 allele exhibited higher BMD at the lumbar spine and femoral neck and experienced less bone loss than ε4 carriers [211]. This observation fits with the differing metabolic profiles of ApoE2 and ApoE4, although mechanistic explanations remain speculative.

Efforts to clarify the biology have highlighted pathways shared between lipid metabolism and skeletal homeostasis. ApoE has been implicated in de novo HDL synthesis and interacts with regulatory networks involving PPARγ, ESR1, vitamin K, and IL‐6, forming part of a gene set associated with both HDL levels and BMD regulation [212, 213, 214]. Yet, direct mechanistic evidence connecting ApoE isoforms to bone physiology is still sparse.

Overall, while some epidemiological data link ApoE, particularly ε4, to lower BMD and increased fracture vulnerability, results across studies remain inconsistent. Environmental and lifestyle factors, dietary patterns, physical activity, and comorbidities add further complexity to interpreting genotype–phenotype relationships. To understand these effects, robust large‐scale studies with careful phenotyping and prospective genotyping will be essential.

### 4.4 Muscle Mass

Skeletal muscle naturally loses mass, strength, and performance with age, a process known as sarcopenia, which in turn raises the risk of cardiovascular disease (CVD), stroke, functional decline, and even mortality. Because muscle weakness, especially reduced handgrip (HG) strength, is such a strong marker of ageing biology, several studies have examined whether genetic factors such as APOE contribute to differences in muscle health across the lifespan. The recurring link between the ε4 allele and poorer muscle traits has led researchers to consider APOE a promising gene for understanding why muscle aging varies so widely between individuals [215].

Experimental models have also shed light on ApoE's role in muscle biology. Earlier work in ApoE KO mice showed that ApoE is present at the neuromuscular junction and is required for healthy mitochondrial function, proper macrophage infiltration, and efficient muscle repair after hindlimb ischemia [216]. Similar impairments were seen after cardiotoxin‐induced injury, where ApoE deficiency slowed muscle regeneration [217]. More recent analyses, however, have painted a more nuanced picture: when muscle tissue from ApoE2, ApoE3, and ApoE4 mice was examined under baseline conditions, no major differences emerged in muscle size, fiber cross‐sectional area, or the abundance of myonuclei or muscle stem cells [218].

Human studies point to clearer isoform‐specific effects during aging. In older adults, ε4 carriers tend to show slower gait speed and poorer performance on chair‐stand tests at baseline compared with ε3 carriers [219]. The ε4 allele is also linked to a greater decline in HG strength between ages 75 and 79 years, and men carrying ε4 appear to lose gait speed more rapidly and face a higher risk of disability over a 3‐year follow‐up period than noncarriers [220, 221].

Altogether, these findings highlight the importance of understanding how APOE genotype shapes muscle aging. Clarifying these genetic influences may eventually help guide strategies to maintain muscle function and counteract sarcopenia as people grow older.

### 4.5 Cardiovascular System

CVD remains the leading cause of mortality worldwide and a major source of long‐term disability. Its prevalence has continued to rise since 1990, driven by aging populations, lifestyle changes, and global increases in exposure to metabolic and environmental risk factors [222].

In this context, ApoE has been identified as a significant genetic factor influencing lipid metabolism and vascular health. Large‐scale population studies have consistently shown that ApoE polymorphisms influence susceptibility to coronary and peripheral vascular disease [16].

Recent work reinforced the idea that the ε4 allele carries a more atherogenic profile, while ε2 may exert a degree of protection. A 2024 study found that ε4 carriers displayed a combination of dyslipidemia and abnormal inflammatory markers strongly linked to elevated CVD risk, whereas ε2 carriers showed a more favorable metabolic signature [223]. Multiple investigations have associated ε4 with atherosclerosis [224] and coronary artery disease [225, 226, 227, 228]. The role of ε2 is less straightforward: while several studies associate ε2 with lower LDL–cholesterol and reduced atherosclerotic risk [224, 229, 230], others link ε2 to dyslipidemia and increased odds of ischemic cardiovascular events [231, 232, 233]. Additional work indicates that APOE variants may also modulate the severity of coronary atherosclerosis  [234, 235, 236], and APOE ε3/ε4 genotypes have been reported as independent risk factors for coronary lesions in patients with hypertension [237].

The connection between APOE and myocardial infarction (MI) has also been widely studied, though with mixed results. One meta‐analysis found a higher prevalence of MI among ε4/ε4 carriers relative to ε3/ε3, but no significant difference for ε2/ε2 [238]. Another meta‐analysis reported the opposite: reduced MI frequency in ε2/ε2, with no difference in ε4/ε4 [239]. A more recent and comprehensive analysis involving more than 28,000 participants concluded that ε2‐involving genotypes tend to be protective, whereas ε4‐involving genotypes (ε4/ε3, ε4/ε4) increase MI risk [240]. Supporting this, ε4 has been repeatedly associated with higher atherosclerotic CVD risk [241] and with premature coronary disease through elevations in total cholesterol and LDL‐C [242].

In individuals with T2DM, APOE genotype may further modulate vascular risk. A cross‐sectional analysis showed that the ε3/ε4 genotype was more common among patients with both T2DM and CVD, and that ε4 carriers without diabetes also showed an elevated CVD prevalence, reinforcing the allele's vascular impact [243].

A large systematic review extended these observations to ischemic stroke, demonstrating a dose‐dependent relationship between APOE genotype and LDL‐C, carotid intima‐media thickness, and stroke incidence. The role of ε2/ε2 remains uncertain and warrants additional study [244]. Beyond genetic variants, ApoE levels within HDL particles may reflect vascular plaque burden: low ApoE–HDL concentrations were strongly associated with early coronary plaque development and an unfavorable cardiometabolic phenotype, suggesting that ApoE content in HDL may be a useful biomarker of CVD severity [245].

More recent large‐scale genetic analyses demonstrate that ApoE contributes to CVD in a vascular‐bed‐specific manner. Variants predicted to increase plasma ApoE and remnant cholesterol were linked to higher risk of peripheral arterial disease, whereas common APOE variants associated with high LDL‐C, TGs, and remnants were primarily linked with ischemic heart disease. Meanwhile, variants associated with low ApoE showed an increased risk of intracerebral vascular disease. These findings underscore that the cardiovascular relevance of ApoE extends beyond classical LDL–cholesterol pathways and involves remnant lipoproteins, TG metabolism, and tissue‐specific vascular vulnerability [246].

Collectively, these data support the idea that APOE genotyping may serve as a useful early‐screening tool for CVD risk. Identifying high‐risk individuals, particularly ε4 carriers, could help guide preventive strategies that incorporate diet, lifestyle, and pharmacological interventions aimed at reducing lipid and inflammation‐driven vascular injury.

### 4.6 Hepatic System

ApoE is expressed in several tissues, with the liver being the main production site. In hepatocytes, it plays a central role in lipoprotein metabolism by serving as a ligand for members of the lipoprotein receptor family, thereby promoting the hepatic uptake and clearance of chylomicron and VLDL remnants. Through these actions, ApoE contributes to the systemic redistribution of cholesterol and TGs and to the regulation of hepatic lipid flux. Human APOE alleles exert broad effects on lipid homeostasis, influencing key aspects of liver metabolism such as VLDL secretion, cholesterol transport, and hepatic lipid export, with clear relevance for metabolic and liver‐related disorders [11, 247].

GWASs and whole‐exome sequencing analyses have identified multiple genetic variants linked to metabolic dysfunction‐associated steatotic liver disease (MASLD) and its inflammatory form, metabolic dysfunction‐associated steatohepatitis (MASH), among which APOE has emerged as a contributing locus. Early observational studies reported limited or inconsistent associations between APOE polymorphisms and fatty liver disease [248, 249, 250]. However, subsequent investigations noted an over‐representation of APOE ε3 homozygotes among patients with MASH [251], a finding later supported by case–control studies suggesting a relative protective effect of the ε4 allele [252, 253]. More recently, large‐scale genome‐ and exome‐wide analyses across different ethnic backgrounds have consistently linked the ε4‐defining SNP rs429358 to variation in liver fat content, liver enzyme levels, and susceptibility to steatotic liver disease [254, 255, 256, 257].

In agreement, rs429358 has been associated with differences in hepatic fat accumulation, circulating liver enzymes, and overall risk of liver disease. In the largest exome‐wide meta‐analysis using computed‐tomography‐derived measures of hepatic steatosis, carriage of the rs429358‐T allele was associated with higher alanine aminotransferase (ALT) levels, increased hepatic steatosis, cirrhosis, elevated circulating TGs, and obesity [258]. Although the underlying mechanisms remain incompletely defined, proposed pathways include isoform‐specific modulation of hepatic mitochondrial function, autophagy, and AMPK–mTOR signaling [259], as well as differences in VLDL secretion, lipid clearance, and immune activation that may contribute to increased susceptibility to steatosis and steatohepatitis in non‐ε4 carriers. Consistent with these observations, a comprehensive review has summarized how APOE polymorphisms influence the risk and progression of several liver diseases. Accumulating evidence suggests that APOE ε4 carriers are relatively protected against chronic hepatitis C virus infection, exhibit slower fibrosis progression, and show reduced susceptibility to alcoholic cirrhosis, MASH, hepatocellular carcinoma (HCC), and hepatitis B virus‐related disease. In contrast, APOE ε3 carriers appear to be at higher risk of developing cirrhosis associated with MASH, MASLD, HCC, or viral hepatitis [260]. While further clinical studies are required, these findings collectively suggest that carriage of the ε4 allele may exert a protective effect against progression of liver disease across different etiologies.

Experimental models further support isoform‐dependent hepatic effects. Mice expressing human APOE ε3 develop more severe hepatic steatosis and liver injury than APOE ε4‐expressing mice when challenged with high‐fat, high‐sugar diets, indicating isoform‐specific susceptibility to hepatic lipid accumulation [261]. In line with this, ApoE‐deficient mice fed a high‐fat diet exhibit exacerbated inflammation, fibrosis, and steatosis compared with wild‐type controls. In this model, the absence of ApoE was associated with mitochondrial dysfunction, reduced AMPK–mTOR signaling, increased oxidative stress, impaired autophagy, and heightened inflammatory responses, collectively aggravating liver injury [259].

More recently, studies using humanized ApoE3 and ApoE4 mouse models demonstrated that the ε4 allele induces extensive alterations in hepatic mitochondrial function and reprograms glucose and lipid metabolism. Similar effects were observed in human induced pluripotent stem cell (iPSC)‐derived hepatocyte‐like cells, where APOE ε4 impaired mitochondrial respiration and promoted a metabolic shift toward glycolysis, resulting in increased lipid accumulation. These findings indicate that APOE genetic variation directly affects hepatic energy metabolism and mitochondrial homeostasis, extending its role beyond lipoprotein trafficking [262]. Moreover, ApoE‐deficient mice display marked disturbances in bile‐acid composition and hepatic cholesterol handling under high‐fat, high‐cholesterol diets, including substantial increases in muricholic and chenodeoxycholic acids, highlighting a critical role for ApoE in maintaining bile‐acid and cholesterol homeostasis in the liver [263].

Supporting these experimental data, a recent Mendelian randomization and network‐analysis study identified elevated APOE expression as significantly associated with increased risk of MASLD and HCC, suggesting that ApoE contributes to disease progression not only through dysregulated cholesterol metabolism but also via interactions within complex regulatory protein networks [264].

Interestingly, despite its well‐established role as a risk factor for NDs, the ε4 allele appears to confer relative protection against steatotic liver disease, steatohepatitis, and even HCC in several studies, compared with ε3. At the same time, rs429358 has also been linked to increased liver fat, elevated ALT, cirrhosis, obesity, and hypertriglyceridemia. These seemingly conflicting findings suggest that ApoE's hepatic effects are strongly context‐dependent and shaped by environmental factors such as diet, obesity, insulin resistance, and immune activation. In addition, different disease endpoints, ranging from simple steatosis to fibrosis, cirrhosis, or cancer, may be differentially influenced by APOE genotype.

Taken together, these data highlight ApoE as a key regulator of hepatic metabolism whose influence extends beyond lipid transport to mitochondrial function, inflammation, and bile‐acid homeostasis. Stratification by APOE genotype may therefore help identify individuals at differential risk for liver disease and support the development of more personalized prevention and treatment strategies across the MASLD‐MASH disease spectrum.

## 5 Translational Implications and Therapeutic Potential

The expanding mechanistic understanding of ApoE biology is now intersecting with a rapidly evolving translational landscape. In parallel with its established relevance to AD, APOE variation is increasingly recognized as a determinant of systemic phenotypes (cardiometabolic risk, vascular integrity, immune responsiveness, and treatment sensitivity) suggesting that ApoE is not merely a risk marker but a modulatory node with clinical leverage. Translational progress therefore depends on two complementary objectives: first, refining how genetic and protein‐level APOE variation is interpreted for risk estimation and patient stratification; second, developing therapeutic strategies that either modify ApoE abundance, structure, lipidation state, or downstream signaling consequences.

This section integrates these themes by outlining (i) the opportunities and constraints of APOE genotyping within precision medicine frameworks, (ii) the current spectrum of ApoE‐ and ApoE4‐informed therapeutic modalities and their mechanistic rationale, and (iii) key challenges that must be addressed to enable clinical implementation, including population diversity, safety liabilities, biomarker alignment, and the need to move beyond brain‐restricted paradigms when systemic effects are implicated.

### 5.1 ApoE Genotyping as a Tool for Personalized Medicine

Before discussing the usefulness of ApoE testing, it is worth clarifying what this type of genetic analysis can and cannot provide. ApoE genotyping is not a diagnostic tool: it cannot confirm whether someone already has a disease, nor can it predict with certainty who will eventually develop one. What it does provide is information about genetic susceptibility, and this represents only one portion of the overall risk. Lifestyle, environmental exposure, and other genetic factors all contribute to health outcomes. Even individuals who carry the high‐risk ε4 allele may never develop the associated conditions. For this reason, it is important to keep expectations realistic when interpreting ApoE test results.

In recent years, several studies have shown that the classical ε2/ε3/ε4 alleles represent only part of the genetic picture. For example, a 2023 analysis sequencing APOE in over 10,000 people (with follow‐up genotyping in nearly 100,000) identified a number of rare, functionally damaging variants present in roughly one out of every 257 individuals in the general population [246]. These variants also contribute to vascular disease risk and highlight the need to look beyond the usual three alleles. Similar findings have emerged in the field of dementia research. By resequencing the APOE gene in more than 10,000 individuals and combining those data with population‐level genotyping, investigators showed that structural variants outside the ε2/ε3/ε4 framework also modulate dementia risk. Interestingly, they found that people who are genetically predisposed to lower ApoE protein levels have a higher dementia risk, whereas genetically higher ApoE levels appear protective [265]. This suggests that protein quantity, not only isoform type, matters. A large study in 2024 further extended this concept by examining both common and rare coding and noncoding variants across more than 13,000 individuals from diverse ancestries. Several additional risk‐associated signals were uncovered, providing a more nuanced genetic landscape of AD that is not fully captured by ε4 alone [266]. In parallel, the largest GWAS to date replicated itself and identified new genomic loci influencing plasma ApoE concentrations. It also examined secondary signals within the ApoE region itself and investigated the relationship between baseline ApoE levels and cognition and future dementia [267].

Genotyping can also reveal heterogeneity among individuals who technically share the same classical genotype. For instance, whole‐exome sequencing studies have identified rare variants that either increase or decrease risk among ε4 carriers, meaning that not all ε4 carriers have the same genetic profile or the same vulnerability [268]. Moreover, two specific missense variants, V236E and R251G, were shown to reduce AD's risk by more than half, further demonstrating that protective variants also exist within this gene [269].

In clinical contexts, APOE testing is becoming increasingly relevant for early risk assessment. For example, ongoing clinical studies enroll cognitively healthy ε4 carriers to determine whether preventive interventions (such as high‐dose DHA supplementation) may delay early pathological changes [270]. Here, the genotype is used to identify people who might benefit from closer monitoring or preventive strategies before symptoms appear.

There is also evidence that different APOE isoforms influence responses to diet and medication. Only ε4 carriers, for instance, significantly lowered serum lipids after consuming plant sterols [271]. In therapeutic contexts, ApoE4 status, especially ε4 homozygosity, has been shown to alter both the effectiveness and risk profile of certain AD treatments, including monoclonal antibodies such as Lecanemab and donanemab [272]. APOE genotype may also affect individual responses to statins and other cardiovascular therapies [229, 273, 274], supporting its use in tailoring cardiovascular therapy.

Taken together, these findings illustrate that APOE genotyping is gradually becoming a useful tool in precision medicine. Knowledge of an individual's APOE profile can help refine risk estimates, guide monitoring strategies, and influence dietary or pharmacological management. It may also be helpful for understanding inherited risk within families. As research continues to expand, particularly in relation to rare variants and protein‐level effects, ApoE genotyping is likely to play an increasingly important role in prevention and tailored medical care.

### 5.2 Therapeutic Strategies

Most therapeutic work on AD still focuses on the treatments we already have or the few that have shown some early promise. For many years, the United States Food and Drug Administration‐approved options were limited to symptomatic drugs, mainly the cholinesterase inhibitors (tacrine, donepezil, galantamine, rivastigmine) and memantine. These drugs help with cognition to some extent, but it is well established that they do not alter the underlying disease process. The more recent introduction of the antiamyloid monoclonal antibodies, aducanumab and lecanemab, represents a major conceptual shift because these agents try to modify pathology. However, the enthusiasm is tempered by the fact that a significant proportion of patients, around one‐third, experience amyloid‐related imaging abnormalities events, including edema and microhemorrhages, which complicates their clinical use [275].

**FIGURE 5:**
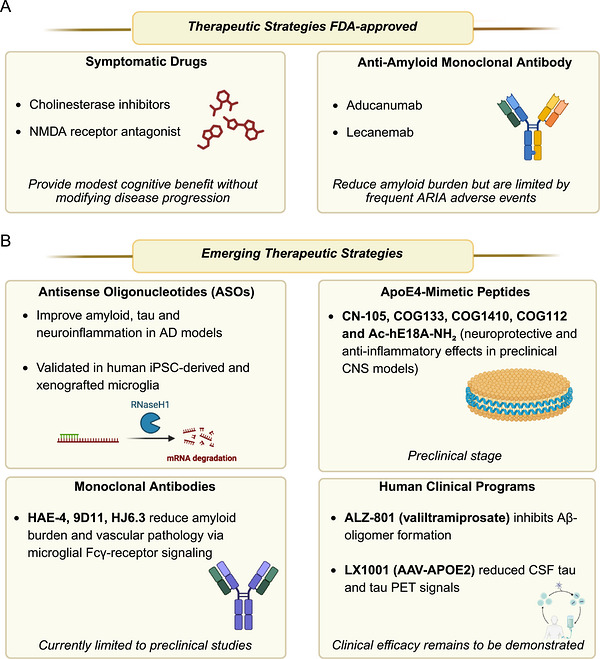
Therapeutic strategies targeting ApoE‐related pathways. United States Food and Drug Administration (US FDA)‐approved treatments include drugs such as cholinesterase inhibitors and NMDA receptor antagonists, which provide modest cognitive benefits without altering disease progression, and antiamyloid monoclonal antibodies (aducanumab and lecanemab), which reduce brain amyloid burden but are limited by the appearance of amyloid‐related imaging abnormalities (ARIA). Emerging approaches targeting ApoE include: (i) antisense oligonucleotides (ASOs), which reduce APOE expression and ameliorate amyloid, tau, and neuroinflammatory pathology in AD preclinical models and in human iPSC‐derived microglia; (ii) ApoE4 mimetic peptides and structural correctors (e.g., CN‐105, COG133, COG1410, COG112, and Ac‐hE18A‐NH_2_), which exhibit neuroprotective and anti‐inflammatory effects in experimental models but remain preclinical for AD; (iii) ApoE‐targeted monoclonal antibodies (HAE‐4, 9D11, and HJ6.3), which reduce amyloid deposition and vascular pathology through Fcγ receptor‐dependent microglial mechanisms in animal models; (iv) gene or molecular therapies in early clinical development, including ALZ‐801 (valiltramiprosate), which inhibits the formation of Aβ oligomers, and LX1001, an AAV‐based APOE2 gene replacement strategy that shows reductions in cerebrospinal fluid tau biomarkers and PET tau signals. Overall, although multiple ApoE‐targeted strategies are under investigation, their long‐term safety and clinical efficacy in AD remain to be established. Image created with Biorender.

Figure 5 and Table 2 provide an integrated overview of current therapeutic strategies targeting ApoE. They also offer a comparative overview of the up‐to‐date pharmacological agents, classified by mechanism of action, developmental phase of preclinical and clinical studies, and experimental limitations.

**TABLE 2:** Overview of current pharmacological agents, clinical trials, and preclinical studies targeting apolipoprotein E, classified by mechanism of action, stage of development, and main limitations.

Therapeutic strategy	Mechanism of action	Key findings/evidence	Stage of development	Limitations	References
Antisense oligonucleotides (ASOs)	Selectively binding to ApoE mRNA, triggering its degradation, and preventing translation into protein	Reduced APOE expression levels in human microglia in vitro and in vivo, resulting in alterations in the phenotypic response of microglia to amyloid‐β pathology. Neuroprotective effects: reducing tau pathology, neurodegeneration, neuroinflammation, mitigating amyloidogenesis, modulating microglial responses and preserved synaptic density in P301S/ApoE4 mice. ≥50% reduction of ApoE mRNA/protein in APP/PS1‐21 ApoE4 mice.	Preclinical (animal studies)	Delivery across the blood–brain barrier; dose optimization; off‐target effects; safety and long‐term effects; risk of immune reactions; limited clinical evidence in humans; specifically target ApoE4 without affecting other isoforms	[276, 277, 278, 279]
ApoE4 mimetic peptides	ApoE mimetics are compounds that mimic key ApoE functions—particularly receptor binding, lipid/cholesterol transport, and anti‐inflammatory/neuroprotective actions—while aiming to neutralize ApoE4‐associated harmful features by reshaping ApoE4 toward an ApoE3/ApoE2‐like conformation.	COG133, COG1410, COG112: neuroprotective; anti‐inflammatory effects; antioxidative effects; reduction neuroinflammation; remyelination and regenerative effects; preserving neurogenesis; reducing amyloid‐ and tau‐like pathology; mitigation AD‐like pathology. AC‑HE18A‑NH2: reduction plasma cholesterol and atherogenic lipoproteins; protection macrophages from oxidized LDL‐induced apoptosis; promoting cholesterol efflux. Chronic administration in APP/PS1ΔE9 mice reduced the load of Aβ aggregation and glial cell activation and improved cognition functions and ApoE levels in the brain. CN‑105: neuroprotective and anti‐inflammatory effects; improved vestibular and later neurocognitive performance; reduced edema and neuroinflammation; promoted neuronal survival. In a mouse model of AD, reduced Aβ pathology and rescued memory deficits; investigated to treat delirium and postoperative cognitive decline and associated neuroinflammation.	Preclinical and clinical trials	Difficulty in transferring effectiveness from animal/cellular models to humans (differences in physiology, disease mechanisms, dosing, etc.); poor stability; short half‐life; poor pharmacokinetics due to sensitivity to proteolytic enzymes; requirement for frequent administration; delivery in effective concentrations to the brain	[275, 280, 281, 282, 283, 284, 285, 286, 287] [288, 289, 290] [291, 292, 293, 294] [295, 296, 297, 298]
Monoclonal antibodies	Some anti‐ApoE antibodies can bind ApoE that is physically embedded within amyloid plaques. Others, for example, HAE‐4, preferentially recognize nonlipidated and aggregated ApoE over the lipidated form found in circulation, and they also bind ApoE within amyloid plaques.	In the APP/PS1 mouse model, HJ6.3 reduced Aβ plaque burden, lowered insoluble Aβ_40_/Aβ_42_, and altered microglial responses around plaques; HAE‐4 treatment reduced parenchymal Aβ and vascular amyloid, improved cerebrovascular function, downregulated inflammation‐related gene expression in the cortex, reduced Aβ‐driven tau seeding/spreading and neuritic dystrophy, and restored neurovascular integrity; in ApoE4 targeted‐replacement mice, 9D11 prevented ApoE4‐driven Aβ accumulation in hippocampal neurons, induced ApoE–IgG complex formation, reversed ApoE4‐associated cognitive deficits, ameliorated‐related pathologies (e.g., hyperphosphorylated tau), and reduced ApoER2 receptor levels.	Preclinical (animal studies)	BBB penetration efficiency; potential disrupting essential ApoE physiological function; difficulty achieving isoform specificity; limited access to intracellular or fragmented ApoE species; risk of immune activation and inflammatory side effects; potential interference with lipid‐sensing and microglial response pathways; steric inaccessibility within plaques; poor reversibility and uncertain clinical translation.	[299, 300]
Small molecule structure correctors	Small molecules, known as “structural correctors,” such as phthalazinone derivatives like “PH002” and “GIND25,” modulate ApoE4 conformation binding to ApoE4 and restore its structure and function to that of ApoE3. This improves lipidation and reduces pathological features, such as reduced Aβ and tau production, in cells that express ApoE4.	In vitro effects: restoration impaired protein trafficking and mobility of ApoE4 in neurons. In human iPSC‐derived neurons, PH002 reduced production/secretion of phosphorylated tau and Aβ species.	In vitro and in vivo studies	Difficulty in achieving brain penetration and adequate target engagement in humans. Complexity of translating protein conformational correction into meaningful clinical outcomes like cognitive improvement.	[301, 302, 303]
Peptidomimetic: PRI‐002 (also known as RD2/“contraloid acetate”)	A d‐enantiomeric peptide that acts on Aβ oligomers, not directly on ApoE: disassembling toxic Aβ oligomers into harmless monomers.	In mouse models: reversal of cognitive deficits and reduction of neurodegenerative markers Patients receiving PRI‐002 performed significantly better on the CERAD word list memory test, suggesting an early cognitive benefit.	Clinical trial (Phase 2)	Early human data showed safety and tolerability, and a hint of cognitive benefit, but no biomarker changes yet.	[304, 305]
Emerging/next‐generation strategies	Gene editing: for example, CRISPR‐based strategies to convert APOE4 allele to APOE3 or APOE2, or to silence ApoE4 selectively, this could “correct” the genetic risk.	Reduction the level of the secreted pathogenic Aβ; attenuation brain Aβ oligomerization and plaque deposition; improvement AD‐related phenotypes	Preclinical	Technically limitations; safety (viral delivery into CNS); off‐target effects; possible immune responses; safety long‐term effects unknown; ethical	[306, 307]
Aβ‐targeted therapies influenced by ApoE genotype (e.g., valiltramiprosate and LX1001)	Oral small‐molecule prodrug that inhibits formation of toxic Aβ42 oligomers, being developed as a precision therapy for APOE4 carriers. While not directly altering ApoE, it's tailored to the higher‐risk ApoE4 genetic background.	Phase 3 in ApoE4/4 early AD: no significant clinical efficacy but slowed brain atrophy	Clinical trial (Phase 3)	No cognitive benefit; unclear ApoE4‐specific mechanism	[311, 312, 313, 314]

Abbreviations: AD: Alzheimer's disease; BBB: blood–brain barrier.

#### 5.2.1 Antisense Oligonucleotides

One area that has attracted much interest is the use of antisense oligonucleotides (ASOs). These molecules are appealing because they can reduce specific RNA transcripts with good precision. Since ApoE isoforms influence tau pathology in different ways, the idea of lowering ApoE4 specifically has received considerable attention. In a P301S/ApoE4 tauopathy model, reducing ApoE4 expression with ASOs led to clear improvements in tau pathology and neurodegeneration [276]. Similar results were obtained in APP/PS1‐21 mice expressing human ApoE3 or ApoE4, where ASO treatment reduced ApoE transcript and protein levels by at least half [277]. A 2024 study demonstrated the efficacy of ASOs in human iPSC‐derived microglia in vitro, as well as their pharmacological activity in vivo in a xenografted microglia model. This study demonstrated that ASOs targeting human microglia can modify their transcriptional profile and their response to amyloid‐β plaques in vivo in an AD model. This work highlights the potential of ASOs as a research tool to study the functions of APOE in neuroinflammation, as well as their potential as a therapeutic modality to interfere with the role of microglia and neuroinflammation in NDs [278].

Overall, ASOs in animal models have demonstrated significant beneficial effects on AD‐related pathologies (amyloid, tau, inflammation) and thus represent a promising therapeutic direction [279]. These findings show that modulating ApoE expression in vivo is feasible, although we are still far from knowing how safe or effective this would be in humans over long periods.

#### 5.2.2 ApoE4 Mimetic Peptides

Another line of research stems from the observation that ApoE4 is more prone to proteolytic cleavage than the other isoforms. This tendency generates specific fragments, some of which may be harmful. Based on this, several groups have been designing short ApoE‐derived peptides, essentially “mimics” of the parts of ApoE4 involved in lipid binding but without the structural liabilities of the full‐length protein [280, 281, 282, 283, 284, 285, 286, 287]. These peptides can cross the BBB and engage relevant receptors, so they have been proposed as possible delivery tools or as direct therapeutic agents. In parallel, there has been steady progress in identifying small molecules that can “correct” the conformation of ApoE4, nudging it toward a structure closer to ApoE3 or ApoE2. These efforts rely heavily on screening libraries and computational modeling, with several compounds showing encouraging activity in cellular assays and in vivo models [288, 289, 290].

Two approaches are generally used for designing new ApoE‐mimetic peptides: by mimicking the primary or secondary structure of native ApoE, or by replicating its biological activity.

Ac‐hE18A‐NH_2_ is an early functional mimetic that links the lipid‐binding 18A helix to ApoE's receptor‐binding domain (RBD 141–150), with terminal modifications for stability, enhancing lipoprotein uptake via HSPGs in hepatocytes [291, 292]. It also exerts a protective effect against apoptosis by reducing cholesterol accumulation [293]. Many additional peptides have been developed using various ApoE regions and sequence modifications to improve pharmacokinetic and pharmacodynamic properties [294].

CN‐105, modeled after the polar helical face of ApoE involved in receptor interaction [295], is small enough to efficiently enter the brain and is widely tested in neurological disease models. In a murine model, CN‐105 improves functional and histological outcomes via modulation of neuroinflammatory pathways [296]. Using a characterized murine model of AD (APP/PS1/APOETR), it was also found that a transient treatment with CN‐105 reduced Aβ pathology and rescued memory deficits in male APP/PS1/APOETR mice when administered early, though not when begun later in disease progression [284]. In intracerebral hemorrhage (ICH), CN‐105 improved vestibular and later neurocognitive performance, reduced edema and neuroinflammation, and promoted neuronal survival [297]. It was also shown to act as an α7‐nAChR antagonist, reducing excitotoxic glutamate release after stroke, and decreased expression of inflammatory and immune‐related genes [297, 298]. A Phase 2 clinical trial (S‐CATCH) is evaluating CN‐105 in acute ICH [287]. Overall, CN‐105 retains neuroprotective and anti‐inflammatory properties and has shown good safety in both preclinical and clinical studies.

Cognosci Inc. has also developed a series of ApoE‐mimetic peptides (COG133, COG1410, and COG112) based on rational drug design, with demonstrated activity in preclinical CNS disease models [283].

However, none of these candidates are ready for clinical testing, and it remains to be seen whether structural correction alone will translate into a robust biological benefit [275, 283].

#### 5.2.3 Monoclonal Antibodies Directed Against ApoE

Since 2012, general anti‐ApoE antibodies (which are not strongly isoform‐selective) have been developed in research. The best‐studied antibodies in the context of neurodegeneration are HJ6.3, HAE‐4, and 9D11. Their properties vary: some bind to all ApoE isoforms, some bind to specific isoforms (e.g. ApoE4), and some bind to aggregated ApoE.

Specifically, HAE‐4 binds both ApoE3 and ApoE4 but has a strong affinity for nonlipidated and aggregated forms, the types most enriched in amyloid plaques. In APPPS1‐21/APOE4 knock‐in mice, HAE‐4 reduced plaque load and appeared to improve aspects of vascular integrity. The antibody crosses the BBB and requires Fcγ‐receptor engagement to work effectively, implying microglial involvement in its mechanism [299]. It also downregulated inflammatory gene expression in microglia and astrocytes, suggesting broader effects on the neuroimmune response [300].

Overall, some of these antibodies, particularly HAE‐4 and 9D11, have demonstrated encouraging preclinical outcomes, such as reducing Aβ pathology, reversing the cognitive and pathological consequences of ApoE4, and eliminating aggregated ApoE from plaques. However, we are still very much in the preclinical phase, and the usual issues of specificity, dosing and chronic safety remain unresolved.

#### 5.2.4 Small Molecule Structure Correctors

The ApoE4 isoform has a unique pathogenic conformation due to an Arg substitution in the Arg61/Glu255 domain interaction. Small molecules that disrupt this intramolecular domain interaction (“structure correctors”) have been identified, which convert ApoE4 to a conformation more similar to ApoE3 in cell assays [301]. There is robust mechanistic, biochemical, and neuronal cell model evidence that structure correctors can ameliorate the deleterious effects of the ApoE4 conformer [302].

In vitro, PH002 rescued dendritic spine development defects and restored functional cellular phenotypes in neuronal cultures expressing ApoE4. In human iPSC‐derived neurons, PH002 reduced production/secretion of phosphorylated tau and Aβ species, effects that were seen only in ApoE4 contexts, supporting the idea that the action is mediated by correcting ApoE4's structure [303]. It was demonstrated that treating neurons derived from iPSCs that expressed ApoE4 with a small‐molecule structure corrector ameliorated the detrimental effects. This shows that correcting the pathogenic conformation of ApoE4 is a viable therapeutic approach for ApoE4‐related AD [289]. In rodent AD models expressing human ApoE4, PH002 and related correctors have been shown to rescue synaptic and mitochondrial phenotypes, but these remain preclinical observations [303]. However, structure correctors identified in preclinical work have not progressed to late‐stage clinical testing.

#### 5.2.5 d‐Enantiomeric Peptide (Peptidomimetic)

PRI‐002 is a peptide consisting entirely of d‐amino acids that is designed to directly disassemble toxic Aβ oligomers into nontoxic monomers via a prion‐like mechanism. Rather than engaging the immune system to clear amyloid, PRI‐002 physically breaks down the toxic oligomeric species thought to disrupt synaptic function in AD.

Preclinical studies in mouse models have shown reversal of cognitive deficits and reduction of neurodegenerative markers following treatment with PRI‐002 [304]. These promising results, coupled with a favorable safety profile, encourage further clinical development of PRI‐002. Indeed, a 2025 study demonstrated that exploratory cognitive measures revealed enhanced performance in memory tests among treated patients. While this study did not demonstrate direct changes in ApoE levels, the cognitive benefit and favorable safety profile support the continued clinical evaluation of PRI‐002 [305]. A multicenter, randomized, placebo‐controlled trial involving patients with MCI and mild AD is currently active across several European countries. Top‐line results are anticipated in mid to late 2026. This study is the first large‐scale efficacy trial and will clarify whether PRI‐002 has disease‐modifying or clinically beneficial effects.

#### 5.2.6 Gene Editing Approaches

Researchers are using CRISPR/Cas9‐based systems with repressor domains to target the APOE4 allele selectively (in vitro and in mouse models), reducing APOE4 mRNA/protein levels without altering the normal APOE3 form. Indeed, gene editing aims to directly alter the APOE gene sequence (e.g., convert APOE4 to a less risky form like APOE3) or modify regulatory elements controlling its expression.

CRISPR–Cas technology has been used in AD research. The mutation leads to increased β‐secretase cleavage of the amyloid beta precursor protein and thus high levels of Aβ. Deleting the mutation in an allele‐specific manner using CRISPR–Cas system reduced the level of the secreted pathogenic Aβ and demonstrated the effectiveness of gene editing as a therapy for familial AD caused by APP dominant mutations [306]. Moreover, converting *APOE* ε4 to *APOE* ε3 was sufficient to attenuate multiple AD‐related pathologies in the neurons, astrocytes, and organoids derived from hiPSCs obtained from a sporadic AD patient  [307].

The use of CRISPR/Cas9‐mediated gene editing approaches can efficiently change the *APOE* genotype and successfully reverse AD‐related phenotypes and impact Aβ aggregation. Altogether, these studies provide a proof of concept for the therapeutic potential of this technology in precision medicine. In summary, the use of CRISPR–Cas gene‐editing technology to target the APOE gene shows potential in ameliorating the pathogenic effects of the ApoE4 isoform. However, despite being scientifically promising, gene editing approaches targeting APOE are still entirely preclinical. None have yet progressed to human trials due to complexities surrounding delivery and safety [306].

#### 5.2.7 ApoE in Non‐Neurological Therapeutic Contexts

Outside the brain, the ApoE genotype has also been shown to influence responses to cardiovascular therapies. A 2025 analysis using UK Biobank and All of US datasets reported that individuals with ε4/ε4 not only had higher cardiovascular and all‐cause mortality during statin therapy but also showed different patterns of LDL‐C, TGs, ApoB, and related biomarkers compared with other genotypes [308]. Studies in Chinese patients with dyslipidemia showed that individuals with ε2 phenotypes responded better to atorvastatin and rosuvastatin in terms of lipid reduction [309]. There are also genotype‐dependent associations between ApoE and FA‐related lipid markers, with ε2 and ε4 groups showing opposite trends [310]. All this points toward a clear role for ApoE in shaping both baseline cardiovascular risk and therapeutic response.

If we step back and look at the field, several therapeutic avenues targeting ApoE or ApoE4 biology are being explored (ASOs, monoclonal antibodies, engineered peptides, structural correctors, and gene therapy among them). Some approaches are more mature than others, but none has yet delivered a definitive clinical benefit in large, rigorous Phase 3 trials. Still, the momentum is increasing, and for the first time, we have multiple strategies that directly address the molecular consequences of ApoE4, rather than simply treating downstream symptoms.

#### 5.2.8 Human Clinical Programs

One of the directly targeted APOE clinical trials in patients with AD is ALZ‐801 (valiltramiprosate). This is an ongoing Phase 3 trial testing an oral antiamyloid agent (ALZ‐801) specifically in patients with early‐stage AD who are homozygous for APOE ε4. This molecule is designed to inhibit β‐amyloid oligomer formation upstream of plaque deposition, in order to slow brain atrophy and improve cognitive function [311, 312, 313]. The efficacy, safety/tolerability, and brain volume effects of valiltramiprosate were evaluated in a Phase 3, randomized, double‐blind, placebo‐controlled, multicenter, 78‐week trial in homozygotes with early symptomatic AD. The APOE4/4 Early AD population did not show significant clinical efficacy at 78 weeks but showed significant brain atrophy slowing [314].

As of late 2025, there are no registered Phase 2 gene therapy or CRISPR‐based clinical trials directly targeting the APOE gene itself. The AAV‐based gene therapy LX1001 is available, but it is currently in Phase 1/2 of an open‐label study. A Phase 2 trial with a placebo control has not yet been conducted. A long‐term safety follow‐up for LX1001 is also registered, but again not a Phase 2 efficacy‐focused trial. This means that next‐generation gene therapy or gene editing approaches aimed at modifying the APOE genotype have not yet progressed into Phase 2 clinical testing in humans.

Overall, ALZ‐801's APOE4 Phase 2 biomarker trial is currently the most relevant Phase 2 study with a therapeutic strategy tailored to individuals carrying the APOE4 allele, though its mechanism targets Aβ rather than APOE directly.

### 5.3 Challenges and Future Directions

With the steady improvement of molecular and imaging technologies, our understanding of ApoE has grown much more quickly than the therapeutic landscape around it. The identification of both common and rare APOE variants is now forcing us to rethink how several diseases begin and why certain individuals are more vulnerable than others. A review of the literature reveals that no single model fully captures the complexity of ApoE biology; findings often differ depending on the system, the disease stage, or even the cell type being studied. This inconsistency is not surprising given that ApoE sits at the crossroads of lipid metabolism, inflammation, neurobiology, and vascular function. However, it does mean that broader, better‐controlled studies are still needed before firm conclusions can be drawn about therapeutic intervention. Still, the general direction is clear enough: understanding how ApoE influences disease mechanisms is likely to be essential if we want to develop more targeted preventive strategies or future interventions.

In this context, APOE genotyping is becoming a useful tool, even if it is not a diagnostic test and should not be interpreted as one. It can help identify people who might benefit from earlier monitoring or lifestyle adjustments, and it is increasingly used in clinical trials to account for the very different trajectories seen in ε2, ε3, and ε4 carriers. Knowing patient's genotype can also guide treatment choices, since several therapies behave differently depending on the isoform background. Genotype information may be relevant as well for counseling families or planning long‐term care. But there are clear limitations. Carrying ε4 raises risk but does not seal patient's fate; ε2 lowers risk but does not eliminate it. Many datasets still come from individuals of European ancestry, so the strength of association varies across populations, and environmental factors can modify risk in ways we are only beginning to quantify. In addition, combining genotyping with advanced biomarkers or imaging is expensive and not feasible in all clinical settings.

Despite all this, it is hard to ignore that APOE genotyping fits naturally into the broader movement toward precision medicine. It illustrates how a single genetic marker, handled carefully and interpreted in context, can help shape more individualized approaches to prevention, prognosis, and treatment. We are still some distance from fully realizing its clinical potential, but the trajectory is unmistakably moving in that direction.

## 6 Conclusion and Prospects

ApoE remains one of the most biologically versatile proteins described in human physiology, mediating not only lipid and cholesterol handling but also processes that extend far beyond classical metabolic regulation. Across different tissues, ApoE influences mitochondrial function, ER stress responses, oxidative, and inflammatory pathways, and even the balance between cell survival and programmed cell death. These functions show clear isoform‐dependent patterns: ApoE4 usually pushes cells toward a proinflammatory, prosenescent, metabolically stressed state, while ApoE2 tends to support more efficient lipid homeostasis, greater stress resilience, and enhanced longevity in many settings. ApoE also shapes immune signaling through its interactions with receptors such as TLR4, LDL receptor family members, and HSPGs, resulting in distinct cytokine and microglial responses across isoforms. Although best known for its role in AD, the APOE genotype is also associated, albeit sometimes inconsistently, with adiposity, fat distribution, cardiovascular markers, and bone and muscle parameters, with effects that depend on age, sex, BMI, and environmental exposures.

Bringing these domains together suggests that ApoE may occupy a central position connecting neurobiology, metabolism and immunity. Studying these intersections may help identify molecular pathways that cut across aging, metabolic disease and neurodegeneration. This is particularly relevant for precision medicine, where ApoE already provides a clear example of how a single gene can shape disease risk, therapeutic response, and prevention strategies. Genotype information is being incorporated into risk stratification models, trial design, and, increasingly, into discussions about personalized lifestyle and therapeutic interventions. At the same time, the literature outside the neurological field remains uneven. Associations between ApoE and metabolic or musculoskeletal traits vary widely between studies, often due to differences in cohort size, ethnicity, the presence of comorbidities, lifestyle factors, and gene–environment interactions. These inconsistencies highlight the need for larger, better‐controlled studies, particularly since metabolic and CVDs frequently coexist with neurological disorders, creating complex bidirectional influences.

Overall, synthesizing ApoE's actions across multiple physiological systems is not simply an academic exercise; it provides an opportunity to understand shared mechanisms that might be targeted therapeutically. Advances in genomics, molecular biology, and high‐resolution biomarker profiling are now making it possible to connect ApoE biology to clinical practice in ways that were not previously feasible. A deeper understanding of how ApoE modifies vulnerability and resilience, whether in the brain, vasculature, adipose tissue or bone, may ultimately support personalized prevention and treatment strategies across a wide range of diseases. As the field moves toward precision health, ApoE is likely to remain a key biological node, both as a biomarker and as a potential therapeutic target.

## Author Contributions

Miriam Frosina and Luca Tirinato suggested the topic of the review. Miriam Frosina, Samantha Maurotti, Alberto Castagna, Tiziana Montalcini, and Luca Tirinato conducted the literature review and wrote the manuscript. Arturo Pujia and Luca Tirinato supervised the preparation and finalization of the manuscript. All authors have read and agreed to submit the manuscript.

## Funding Information

This work was also supported by the European Union “Next Generation EU” through the National Plan of Recovery and Resilience (PNRR), which financed the project PRIN PNRR titled “Peritumoral Adipose Tissue Lipid Trafficking Investigation through Raman spectroscopy” (CUP F53D23011350001; project code P2022YAKJY).

## Conflicts of Interest

The authors declare no conflicts of interest.

## Ethics Statement

The authors have nothing to report.

## Data Availability

All data are included in the paper.
